# Electrochemical Interlayer Expansion and Dual Redox Activation for Fast Mg-Ion Transport and High Capacity in Quasi-1D TiS_**3**_

**DOI:** 10.1021/acssuschemeng.5c09578

**Published:** 2026-01-09

**Authors:** Pengcheng Jing, Atsushi Inoishi, Chengcheng Zhao, Eiichi Kobayashi, Yisong Han, Duncan H. Gregory

**Affiliations:** † WestCHEM, School of Chemistry, Joseph Black Building, 3526 University of Glasgow, Glasgow G12 8QQ, U.K.; ‡ Institute for Materials Chemistry and Engineering, 12923 Kyushu University, Kasuga-Koen 6-1, Kasuga, Fukuoka 816-8580, Japan; § 133789 Kyushu Synchrotron Light Research Center, 8-7 Yayoigaoka, Tosu, Saga 841-0005, Japan; ∥ Department of Physics, 2707 University of Warwick, Coventry CV4 7AL, U.K.

**Keywords:** magnesium-ion batteries (MIBs), titanium trisulfide (TiS_3_), interlayer expansion, 1-butyl-1-methylpyrrolidinium, dual redox

## Abstract

Magnesium ion batteries (MIBs) offer promising solutions for next-generation sustainable energy storage systems owing to their intrinsic safety and cost-effectiveness, yet their development is hindered by the scarcity of high-capacity cathode materials, primarily due to poor magnesium ion transport and a limited number of electrochemically active sites. Here, we report a significant performance breakthrough in a structurally and electrochemically distinct, underexplored quasi-1D pseudolayered titanium trisulfide (TiS_3_) cathode through interlayer engineering and exploitation of dual cationic/anionic redox chemistry. *In operando* and *ex situ* characterization reveal that interlayer expansion, induced by the intercalation of 1-butyl-1-methylpyrrolidinium (BMPyrr^+^), weakens electrostatic interactions within the sulfide sublattice, enhances magnesium ion diffusion kinetics, and increases accessible redox sites. These modifications activate reversible Ti^4+^/Ti^3+^ and S_2_^2–^/S^2–^ redox couples, complemented by nanosizing-induced pseudocapacitance, synergistically underpinning the exceptional electrochemical performance. As a result, the expanded TiS_3_ cathode delivers outstanding reversible capacities (up to 300 mA h g^–1^ at 100 mA g^–1^), excellent rate performance (181 mA h g^–1^ at 1000 mA g^–1^), and long-term cycling stability, surpassing its pristine counterpart and many state-of-the-art MIB cathodes. This work underscores the combined role of interlayer engineering and dual-ion redox chemistry in advancing multivalent energy storage and introduces pseudolayered TiS_3_ as a new structural platform beyond conventional layered sulfides.

## Graphical abstract

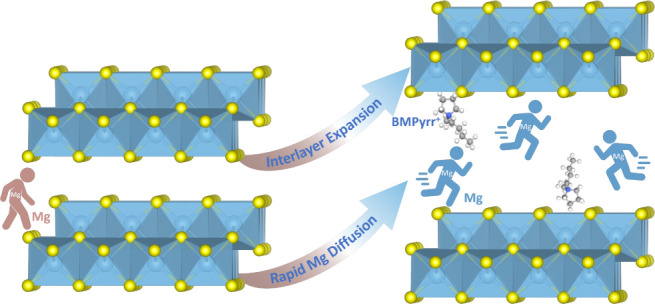

## 1 Introduction

Lithium ion batteries (LIBs) dominate the energy storage landscape for portable electronics and electric vehicles due to their high energy density and long cycle life.^1–3^ However, further improvements in energy density are approaching practical limits, while safety risks (e.g., flammable electrolytes and thermal runaway) and concerns about lithium resource scarcity have spurred the search for alternative chemistries.^4^ Magnesium-ion batteries (MIBs) have emerged as a promising alternative owing to the dendrite-free magnesium metal anode with a high theoretical capacity (2205 mA h g^–1^ vs ∼372 mA h g^–1^ for graphite in LIBs), combined with the abundance and low cost of magnesium.^5,6^ Despite these advantages, the progress of MIBs is hindered by the lack of high-performance cathode materials, primarily due to poor Mg^2+^ diffusion and the limited number of accessible redox sites.

Chalcogenide cathodes, particularly sulfides and selenides, have shown promise for MIBs because the softer S^2–^ or Se^2–^ anions interact less strongly with Mg^2+^ than O^2–^ in oxides, facilitating ion migration and enhancing capacity.^7–11^ Early breakthroughs include the Chevrel phase Mo_6_S_8_, which offers excellent cycling stability but a moderate capacity of *∼*74 mA h g^–1^.^12^ A considerable proportion of subsequent research expanded to layered transition metal dichalcogenides (e.g., MoS_2_, TiS_2_, VS_2_, WSe_2_)^13–16^ and 1D chain-like compounds such as VS_4_.^17^ In these conventional 2D layered systems, Mg^2+^ intercalation is often limited by strong electrostatic interactions and the reliance on transition-metal-centered cationic redox, which restricts multielectron transfer and overall capacity. By contrast, 1D chalcogenides like VS_4_ contain disulfide (S_2_^2–^) moieties that enable both cationic and anionic redox, alleviating electrostatic constraints and providing access to higher capacities.^17,18^

Transition metal trisulfides (TMS_3_) combine the merits of 1D and 2D architectures.^19^ Their quasi-1D chains assemble into pseudolayered structures with van der Waals (vdW) gaps, offering 2D ion diffusion channels while maintaining fast electronic conduction along the chains.^19,20^ Among them, monoclinic TiS_3_ stands out with its unique dual redox-active centers (Ti^4+^ and S_2_^2–^) and anisotropic structure. Although TiS_3_ has been investigated in lithium and sodium-ion batteries, its electrochemical behavior in MIBs remains underexplored.^21–23^ Previously reported capacities, ∼84 mA h g^–1^ at a low current density of 10 mA g^–1^,^24^ are far below the theoretical values (372 mA h g^–1^ assuming two-electron transfer *via* S_2_^2–^/S^2–^, or 559 mA h g^–1^ with three-electron transfer involving both S_2_^2–^/S^2–^ and Ti^4+^/Ti^3+^ redox), indicating substantial untapped potential.

Interlayer engineering is a powerful strategy to enhance ion diffusion and activate redox reactions in (pseudo)­layered materials.^25^ The insertion of guest species (e.g., organic cations or amines) has been demonstrated to expand interplanar spacing, lower diffusion barriers, and increase the number of active sites.^16,26,27^ For example, BMPyrr^+^ intercalation has been shown to significantly enhance the performance of TiS_2_ and VS_4_ electrodes.^28,29^ However, TiS_3_, with its quasi-1D pseudolayered structure and dual redox capability, has not been systematically studied under such interlayer modification.

In this work, we demonstrate a BMPyrr^+^-expanded pseudolayered TiS_3_ cathode that delivers a high reversible capacity of up to 300 mA h g^–1^ and excellent rate performance, outperforming pristine TiS_3_ and many state-of-the-art MIB cathodes. *In operando* and *ex situ* analyses reveal that BMPyrr^+^ intercalation enlarges the vdW gaps, mitigates electrostatic interactions, and enables reversible dual cationic (Ti^4+^/Ti^3+^) and anionic (S_2_^2–^/S^2–^) redox reactions, complemented by nanosizing-induced pseudocapacitance. These findings highlight TiS_3_ as a structurally and electrochemically distinct platform beyond conventional layered sulfides and provide new design principles for multivalent energy storage materials.

## 2 Experimental Section

All experiments described below were conducted at room temperature (RT), unless explicitly stated otherwise.

### 2.1 Physical Vapor Transport (PVT) Synthesis of TiS_3_ and TiS_2_

TiS_3_ was synthesized *via* a simple PVT method. Typically, a slightly overstoichiometric mixture of titanium (99.5%, 325 mesh, Alfa Aesar) and sulfur (99.98%, Sigma-Aldrich) powders (molar ratio 1:3.15; 0.239 g of Ti, 0.504 g of S) was thoroughly ground in an Ar-filled glovebox, sealed in a quartz tube under a vacuum of *ca*. 10^–4^ mbar, and subjected to heating in a horizontal tube furnace (CARBOLITE, MTF 12/38/400) with a heating profile: ramped at 200 °C h^–1^ to 115 °C and dwelled for 3 h, and then heated at 200 °C h^–1^ to 550 °C for 72 h. After cooling to RT, the product was collected, ground, and stored under Ar. TiS_2_ was synthesized similarly, using a 1:2.1 molar ratio of titanium and sulfur powders and a heating profile with a 700 °C dwell for 15 h.

### 2.2 Material Characterization

TiS_3_ samples before and after cycling were characterized using powder X-ray diffraction (PXRD), Raman spectroscopy, thermogravimetric analysis (TGA), X-ray photoelectron spectroscopy (XPS), X-ray absorption spectroscopy (XAS), scanning electron microscope (SEM), and transmission electron microscope (TEM).

PXRD of the as-synthesized TiS_3_ powder was performed in Bragg–Brentano geometry (flat plate, reflection) over a 2*θ* range of 2° or 5°–70° with a step size of 0.0175° and a scan rate of 0.083° s^–1^ on a Rigaku MiniFlex diffractometer equipped with an unmonochromated Cu K_α_ X-ray source operated at 40 kV and 40 mA, and in transmission geometry (capillary, 5°–80°, 0.0131°, 0.016° s^–1^) on a PANalytical Empyrean diffractometer with an unmonochromated Cu K_α_ X-ray radiation operated at 45 kV and 40 mA. *In operando* and *ex situ* PXRD patterns were collected using Bragg–Brentano (2° or 5°–60°, 0.0263°, 0.19° s^–1^) and capillary transmission (5°–70°, 0.0131°, 0.016° s^–1^) geometries on the PANalytical Empyrean diffractometer. A bespoke CR2032 coin cell was designed for the *in operando* PXRD experiments, with a 7 μm-thick Kapton window (8 mm diameter, sealed with glue and PVDF) at the center of the positive case.

Raman spectra were collected using a LabRAM HR spectrometer with a green laser (wavelength of 532 nm). TGA was conducted on a TA Instruments SDT Q600 instrument under a 2% O_2_/98% Ar gas atmosphere (a flow of 100 mL min^–1^, used as an oxidizing environment) from RT to 550 °C at a rate of 10 °C min^–1^. The elements of interest and their oxidation states in the samples were studied using a JSPS-9010MC/IV (JEOL) X-ray photoelectron spectrometer with an Mg K_α_ X-ray source. Samples were transferred *via* a custom-made transfer vessel (JEOL) to reduce oxidation. High-resolution XPS spectra were fitted according to Conny and Powell, employing dual Gaussian–Lorentzian functions to precisely fit peaks and binding energies.^30,31^ For the collection of *ex situ* XAS spectra, three distinct samples were prepared: the uncycled TiS_3_ powder, cycled TiS_3_ electrodes (3 mm × 5 mm; on Ni current collector), and an MgS powder standard. Each sample was adhered onto carbon conductive tape and transferred to a custom-made transfer vessel in the argon-filled glovebox and transport to the beamline for measurements without exposure to air. The XAS measurements for the Ti L-edges (440 to 480 eV) were performed at Beamline 12 located at the SAGA-LS (Japan), and S K-edges (2450 to 2530 eV) at BL2A beamline of the UVSOR Synchrotron Facility, Institute for Molecular Science (Japan). These spectra were used to investigate the oxidation states and coordination environment of titanium and sulfur within the TiS_3_ cathodes. The spectra were collected in total electron yield mode and calibrated using the S K-edge of an Li_2_S spectrum. The morphologies and spatially resolved elemental composition of TiS_3_ were characterized using a combination of SEM (TESCAN CLARA SEM, 15 kV, equipped with an Oxford Instruments UltimMax 65 EDS operated at 15 kV) and TEM (JEOL 2100plus, operated at 200 kV) instruments.

### 2.3 Electrochemical Measurements

Electrochemical testing of TiS_3_ electrodes was conducted in CR2032 coin cells. Magnesium metal foil chips (0.2 mm thickness, diameter 15 mm, 99.5%, sourced from Huabei Magnesium Processing Plant) were used as the anodes. The cathodes were prepared by casting slurries of 0.07 g of active material, 0.02 g of conductive carbon (carbon black, 99%, Alfa Aesar), and 0.01 g of polyvinyldifluorine (PVDF, 98%, average molecular weight ∼534,000, Sigma-Aldrich) binder in *ca.* 0.5 mL of *N*-methyl pyrrolidone (anhydrous, 99.5%, Sigma-Aldrich) onto Ni foam chips (99.8%, 12 mm diameter, 1 mm thickness, areal density of 280–420 g m^–2^, Saibo Electrochemistry). Subsequently, the cathodes were dried under static vacuum (0.1 mbar) in a drying oven at 60 °C overnight. A 0.4 M “All-phenyl complex” (APC; 10 mL) was used as the *unmodified* electrolyte. It was prepared by dropwise adding 4.0 mL of a solution of phenyl magnesium chloride (PhMgCl, 2.0 M in tetrahydrofuran (THF), Sigma-Aldrich) into 0.534 g of AlCl_3_ (ultra dry, 99.99%, Thermo Fisher Scientific) in 6 mL of THF (≥99.9%, anhydrous, inhibitor-free, Sigma-Aldrich) under magnetic stirring. An additive-containing electrolyte was prepared by adding 0.089 g of 1-butyl-1-methylpyrrolidinium chloride (BMPyrrCl, 99%, Aladdin) into 2 mL of the *unmodified* electrolyte.

Galvanostatic discharge–charge tests, as well as galvanostatic intermittent titration technique (GITT) experiments, were conducted over 0.01–2.0 V using a LAND CT2001A battery test system. For GITT experiments, the test batteries were discharged for 600 s at a current density of 50 mA g^–1^, followed by a relaxation period (no current applied) of 1200 s. These discharging/relaxation or charging/relaxation steps were repeated until the discharging limit of 0.01 V or charging limit of 2.0 V was reached. The details of how these data were used to calculate diffusion coefficients are provided in Equation S4 and Figure S17. Cyclic voltammetry (CV; 0.2–0.8 mV s^–1^, scan from open-circuit voltage to 0.01 V, followed by reverse scan to 2.0 V) and electrochemical impedance spectroscopy (EIS; 100 kHz to 0.01 Hz, 10 mV amplitude) measurements were performed using a PalmSens4 potentiostat at RT. The obtained Nyquist plots were fitted using AfterMath software.

## 3 Results and Discussion

As illustrated in Scheme 1, the Mg-TiS_3_ cell comprises a Mg metal anode, a TiS_3_ cathode, and a glass fiber separator soaked with liquid electrolyte. The pristine TiS_3_ cathode, with its narrow interlayer spacing, suffers from strong electrostatic interactions with magnesium ions, which hinder magnesium ion diffusion and result in low reversible capacity. Expanding the interlayer spacing is expected to alleviate these limitations. Enlarged vdW channels can weaken intercalant-lattice interactions and lower the energy barriers for magnesium ion intercalation and diffusion, thereby activating ion-storage sites that are inaccessible within the pristine structure. In addition, the presence of unique S_2_^2–^ moieties in TiS_3_ offers extra electron transfer pathways for contributing to higher capacity. In the present study, bulky organic BMPyrr^+^ cations are employed to achieve the expanded structure. This additive has been demonstrated to be effective and broadly applicable across various hosts, including (pseudo)­layered sulfides and oxides as well as 1D chain-structured sulfides.^28,29,32,33^

**Scheme 1:**
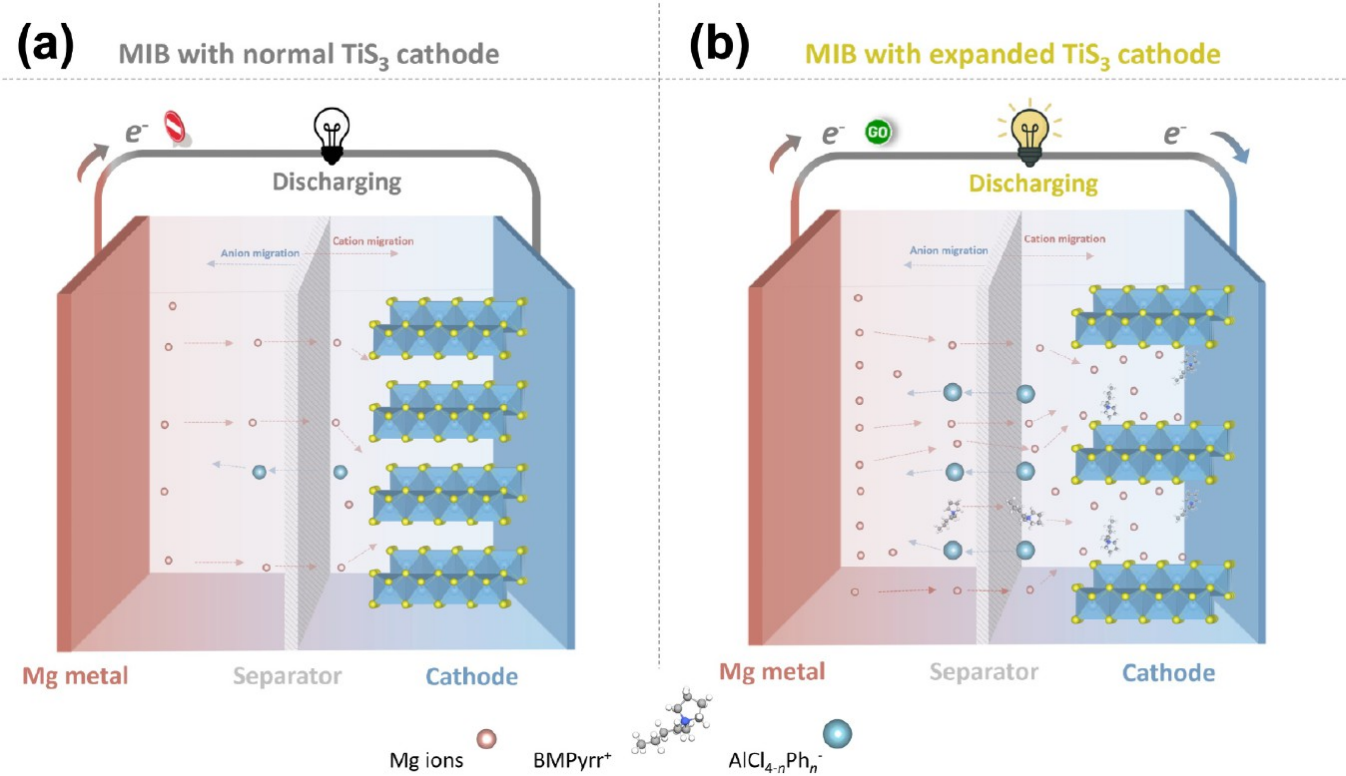
Schematic Illustration of MIB Cells Employing Two TiS_3_ Cathode Configurations: (a) Pristine TiS_3_ with Poor Magnesium Ion Diffusion Kinetics and Limited Ion-Storage Capacity, and (b) Expanded TiS_3_ Designed to Enhance Magnesium ion Diffusion and Increase Accessible Sites^a^

a Both schematics depict the cells in the discharging state.

The TiS_3_ material was synthesized by a physical vapor transport (PVT) method (details in Supporting Information). The phase purity of the as-prepared TiS_3_ was confirmed by PXRD (Figure S1), which matches the monoclinic phase (PDF—80-0924; space group P2_1_/*m*). Subsequently, Rietveld refinement against PXRD data collected in transmission geometry (capillary mode) provided precise structural parameters (Figure 1a, Tables S1 and S2), yielding *a* = 4.9702­(3) Å, *b* = 3.4020­(1) Å, *c* = 8.8046­(6) Å, and *β* = 97.64(1)°, consistent with previously reported values.^34,35^ The refined crystal structure (Figure 1b–d) highlights its pseudolayered configuration composed of 1D [TiS_6_] trigonal prism chains. As shown in Figure 1b,c, the face-sharing [TiS_6_] trigonal prisms form chains along the *b*-axis *via* strong Ti–S polar covalent bonds (light orange bands).^36^ Along the *a*-axis, Ti^4+^ cations in one chain interact weakly with S^2–^ anions of neighboring chains (Figure 1c), creating discrete pseudolayers.^21,36–38^ Viewed along the *a*-axis (Figure 1d), these pseudolayers stack along the *c*-axis *via* vdW forces, forming bulk crystals. The interlayer spacing of the (001) plane is *∼*0.873 nm, defined as the distance between the central planes of adjacent pseudolayers, while the vdW gap (between the outermost S layers) is ∼0.32 nm (red dashed lines in Figure 1d).^39^

**Figure 1:**
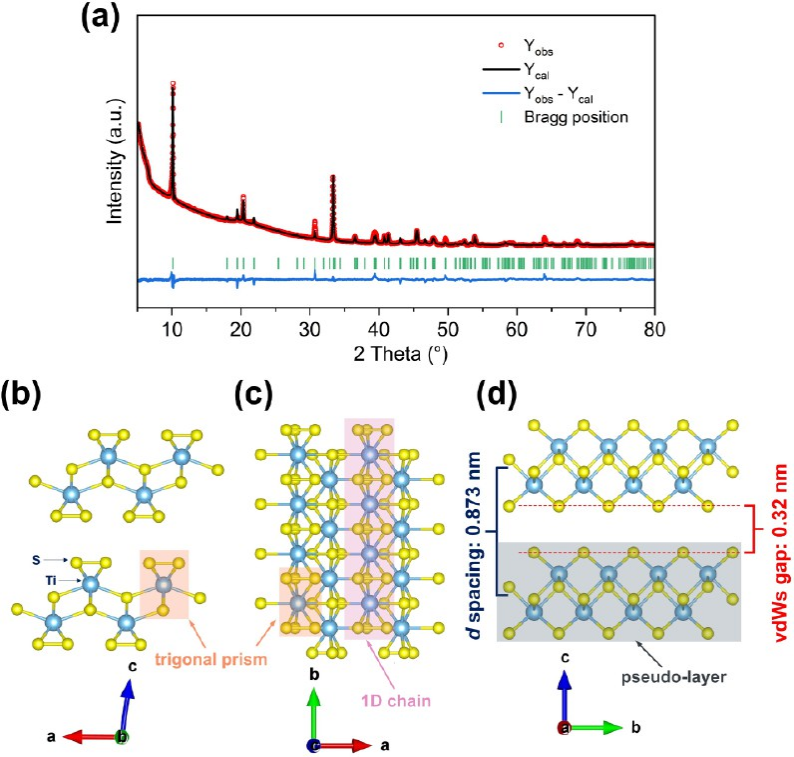
(a) Refined PXRD profile plot for the as-synthesized TiS_3_ powder. (b)–(d) Crystal structure of TiS_3_ projected onto the *ac*, *ab*, and *bc* planes, respectively, as visualized by Vesta software.^40^

Raman spectroscopy of the as-prepared TiS_3_ sample (Figure 2a) reveals four prominent peaks at 172 cm^–1^, 296 cm^–1^, 367 cm^–1^, and 556 cm^–1^, corresponding to A_g_^rigid^, two types of A_g_^internal^, and A_g_^S–S^ vibrational modes, respectively, consistent with the literature.^41^ Specifically, the A_g_^rigid^ mode at 172 cm^–1^ originates from the out-of-plane rigid vibration of individual TiS_3_ chains, while the A_g_^internal^ modes at 296 cm^–1^ and 367 cm^–1^ are associated with two distinct in-plane degenerate vibrations involving Ti–S bonds and bridging/pairing S atoms in neighboring [TiS_6_] prisms (Figure 2b). The A_g_^S–S^ peak at 556 cm^–1^ originates from the in-plane polysulfide vibrations.

**Figure 2:**
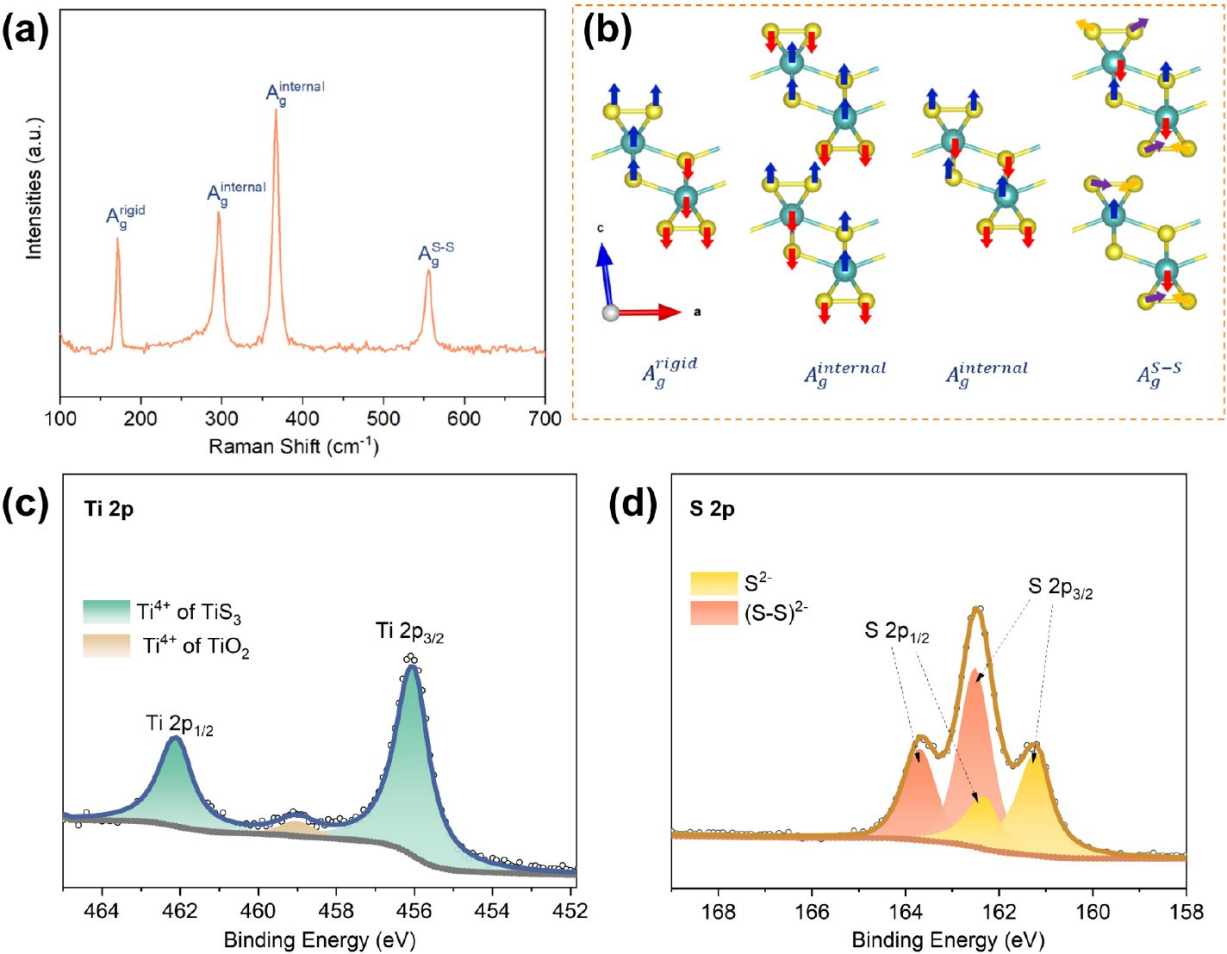
(a) Raman spectrum, and (b) corresponding vibration mode illustration of the TiS_3_ compound.^41^ (b) Reproduced from ref 41. Available under a CC BY-NC 4.0 license. Copyright © 2016, Kedi Wu et al. High-resolution XPS spectra in the (c) Ti 2p and (d) S 2p regions for the as-made TiS_3_ compound.

XPS was used to investigate the oxidation states of Ti and S in the as-synthesized TiS_3_. The high-resolution Ti 2p spectrum (Figure 2c) exhibits three peaks: the doublet at 456.1 eV (Ti 2p_3/2_) and 462.1 eV (Ti 2p_1/2_) are assigned to Ti^4+^ in TiS_3_,^42^ whereas a weaker peak at 459.0 eV suggests trace Ti^4+^ from TiO_2_ surface oxidation layer.^43,44^ In the S 2p region (Figure 2d), two doublets are fitted: peaks at 161.2 eV (S 2p_3/2_) and 162.4 eV (S 2p_1/2_) are assigned to S^2–^ species, while those at 162.5 and 163.7 eV correspond to disulfide (S_2_^2–^) species.^42^

The morphology and composition of the as-prepared TiS_3_ were examined using SEM-EDS and TEM analyses. As shown in Figure 3a–c, low- and high-magnification SEM images reveal that TiS_3_ forms aggregated micro-to-nano size ribbon- or platelet-like particles with lengths of 1–10 μm and widths of 1–3 μm. These particles are loosely aggregated, enabling individual TiS_3_ particles to disperse upon ultrasonic treatment in organic solvents such as ethanol.^19,45^ EDS elemental mapping (Figure 3d,e) confirms the homogeneous distribution of Ti and S across the TiS_3_ micro/nano platelets. Quantitative EDS analysis suggests an S:Ti atomic ratio of 2.82:1, slightly lower than the expected 3:1 ratio. This deviation likely arises from (a) surface oxidation, with the release of volatile sulfur species (e.g., SO_2_ and/or H_2_S), consistent with XPS results; and (b) the known underestimation of elements such as sulfur due to X-ray absorption in EDS. To verify the bulk stoichiometry, TGA analysis was employed on *∼*15 mg of TiS_3_ powder. Oxidation up to 550 °C in an O_2_/Ar atmosphere yielded single-phase TiO_2_, confirmed by PXRD (Figure S2). The calculated S:Ti atomic ratio from TGA data is 2.95 ± 0.01, closely matching the expected stoichiometry (see Supporting Information). TEM observations (Figure 3f,g) further confirm the micro-to-nanoscale ribbon- or platelet-like morphology, with fine ribbons/platelets interspersed among larger particles. HRTEM imaging (Figure 3h), analyzed using DigitalMicrograph software,^46^ reveals well-defined lattice fringes with an interplanar spacing of 0.87(1) nm, corresponding to the (001) crystal plane of monoclinic TiS_3_ and representing the pseudolayer separation derived from the refined crystal structure. These combined structural and compositional analyses validate the high crystalline quality and near-stoichiometric composition of TiS_3_, while its ribbon-like morphology with open edges is expected to facilitate ion diffusion and subsequent interlayer modifications.

**Figure 3:**
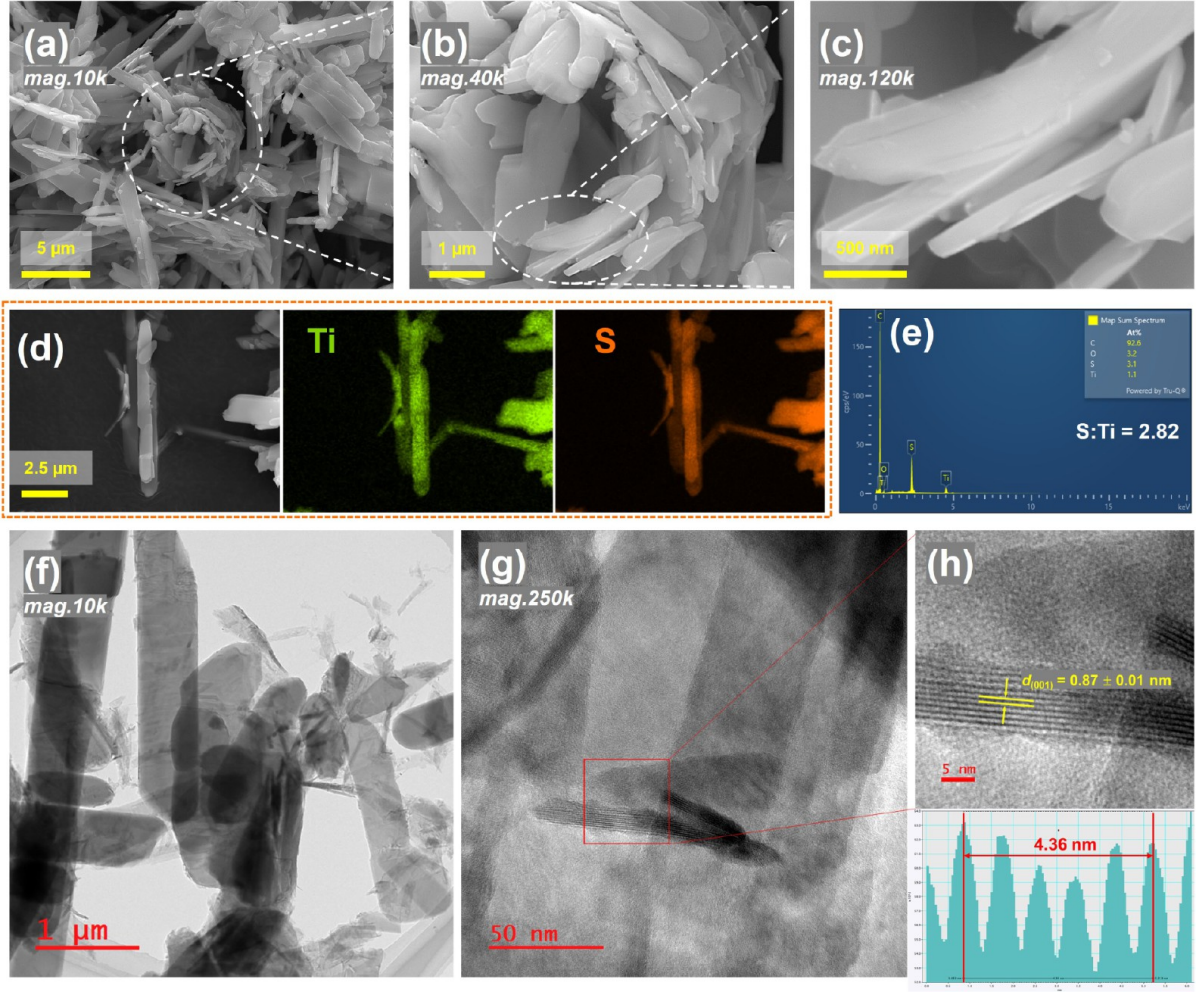
Electron microscopic characterization of the as-synthesized TiS_3_: (a)–(c) SEM images of a part of the as-synthesized TiS_3_ compound at different magnifications of 10k, 40k, and 120k. (d) SEM image of a selected region and corresponding Ti and S elemental EDS maps, and (e) elemental mapping EDS spectrum from the whole region shown in (d). (f) Low-magnification (×10k) and (g) high-magnification (×250k) TEM images, and (h) HRTEM image with measurement graph presented.

The electrochemical activity of TiS_3_ electrodes was evaluated using galvanostatic (dis)­charge and cyclic voltammetry (CV) measurements. In the *unmodified* APC electrolyte, the TiS_3_ electrode displays poor magnesium storage, with the first discharge showing a sloping profile at ∼0.5 V and delivering a low capacity of 32 mA h g^–1^, which rapidly declines to 10 mA h g^–1^ in subsequent cycles (Figure S3a). By contrast, the addition of BMPyrrCl markedly activates the TiS_3_ electrode. As illustrated in Figure 4a, the first discharge exhibits two short slopes (0.98–1.16 V and 0.56–0.70 V) and a distinct plateau near 0.54 V, delivering a high capacity of 575 mA h g^–1^. This significant enhancement suggests that bulk organic BMPyrr^+^ cations likely intercalate into the TiS_3_ lattice, expanding the interlayer spacing and enabling faster magnesium ion diffusion,^28,32^ while simultaneously activating reversible dual redox processes involving Ti^4+^/Ti^3+^ and S_2_^2–^/S^2–^. The slightly higher first discharge capacity relative to the theoretical value may be attributed to partial decomposition, parasitic reactions with the electrolyte, and additional surface charge storage induced by *in situ* nanosizing (discussed later). The first charge delivers a reversible capacity of 388 mA h g^–1^ with a sloping profile, and subsequent cycles show increasingly similar and overlapping curves, indicating structural stabilization and enhanced electrochemical reversibility. Notably, limiting the initial discharge to 0.35 V yields a lower activation capacity (∼350 mA h g^–1^) and a lower long-term capacity (∼191 mA h g^–1^) than discharging to 0.01 V (SI Figure S3c,d). This demonstrates that deeper activation facilitates complete BMPyrr^+^-mediated structural opening and diffusion-channel formation for optimal magnesium storage.

**Figure 4:**
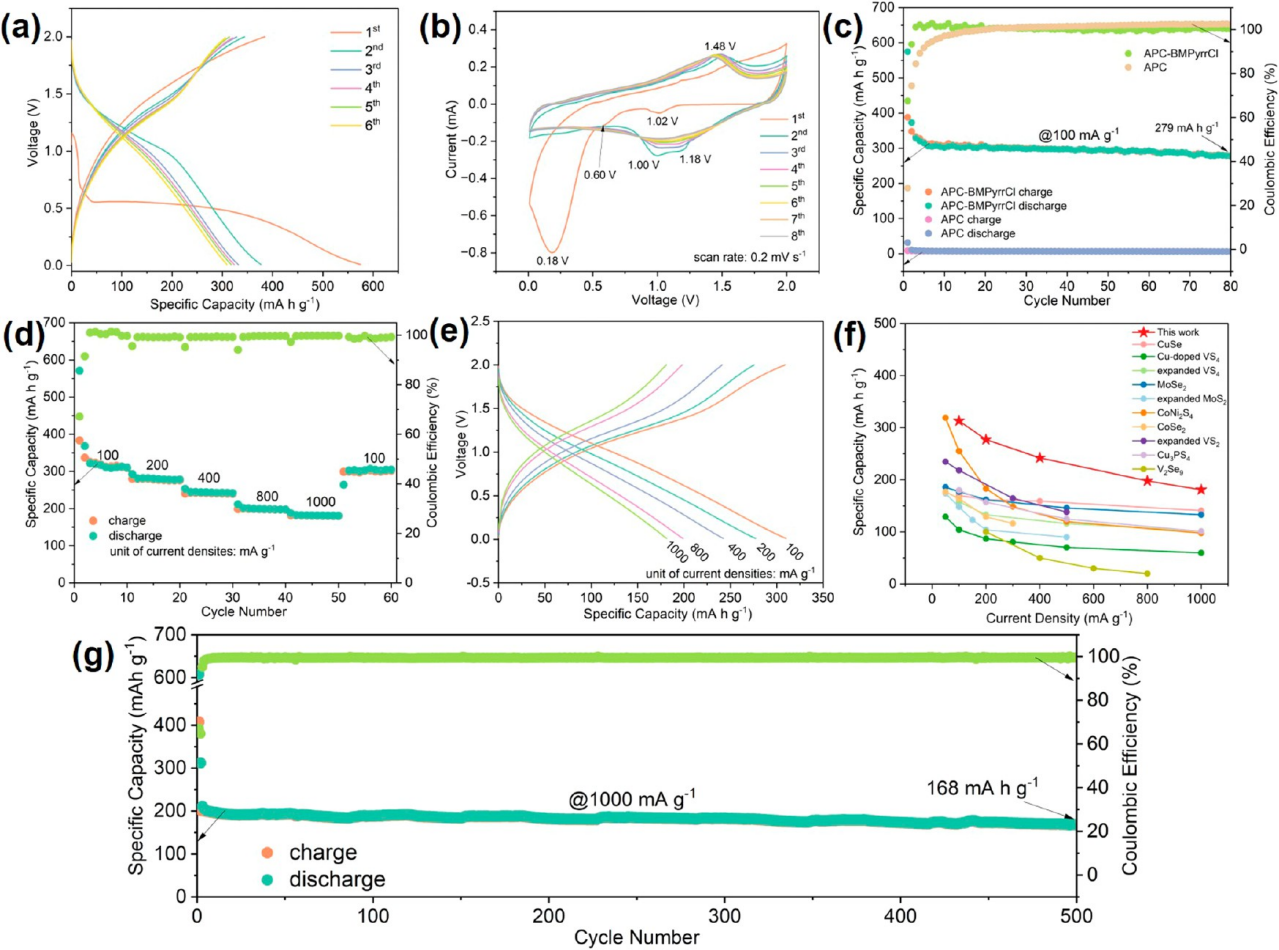
Electrochemical properties and performance of the TiS_3_ electrodes in (−)­Mg|APC-BMPyrrCl|TiS_3_(+) full cell: (a) initial six cycles of galvanostatic (dis)­charge curves at a current density of 100 mA g^–1^ and (b) CV curves from the first eight cycles measured at a scan rate of 0.2 mV s^–1^. (c) Galvanostatic (dis)­charge cycling performance at a current density of 100 mA g^–1^. (d) Rate performance at various current densities. (e) Selected (dis)­charge curves taken at different current densities from the measurements in (d). (f) Capacity comparison at selected current densities for various chalcogenide cathodes. (g) Long-term cycle performance at a high current density of 1000 mA g^–1^.

CV profiles at a scan rate of 0.2 mV s^–1^ further confirm the additive’s role. In pure APC (Figure S3b), the TiS_3_ electrode exhibits two weak, irreversible cathodic peaks at ∼0.63 and 0.50 V, with minimal activity thereafter. With BMPyrrCl (Figure 4b), pronounced cathodic peaks emerge in the first scan, including (a) two small peaks at ∼1.02 and 0.60 V, likely associated with BMPyrr^+^ intercalation and structural expansion that lower magnesium ion diffusion barriers; and (b) a strong peak at ∼0.18 V (0.5–0.01 V), associated with cointercalation of BMPyrr^+^ and magnesium ions and the initiation of dual redox processes. The anodic sweep shows a broad slope (1.25–2.0 V) in which small, broad "bump" peaks (e.g., 1.55 and 1.81 V) overlap with each other, indicating partial reversibility in the first cycle due to trapped BMPyrr^+^ “pillars” and magnesium ions. In the second cycle, cathodic peaks shift to ∼1.0 and 1.18 V, and a merged anodic peak appears at ∼1.48 V, likely corresponding to the S_2_^2–^/S^2–^ and Ti^4+^/Ti^3+^ couples.^47,48^ Subsequent CV scans show increasingly similar profiles in which anodic/cathodic peak separation becomes smaller, confirming improved reversibility with cycling.

Galvanostatic cycling performance of TiS_3_ electrodes in BMPyrr^+^-containing and *unmodified* APC electrolytes was compared at a current density of 100 mA g^–1^. As shown in Figure 4a,c, the TiS_3_ electrode in BMPyrr^+^-containing electrolyte achieves high discharge and charge capacities of 575 mA h g^–1^ and 388 mA h g^–1^, respectively, in the first cycle, corresponding to an initial Coulombic efficiency of 67.5%. After an initial drop, the capacity stabilizes and retains a high value of 279 mA h g^–1^ after 80 cycles. In contrast, the electrode in pure APC remains nearly inactive. Notably, an “expanded” TiS_3_ electrode, predischarged in BMPyrr^+^-containing electrolyte and then cycled in pure APC, exhibits a maximum capacity of 291 mA h g^–1^ and retains 236 mA h g^–1^ after 80 cycles, with (dis)­charge and differential capacity (DC) profiles resembling those in BMPyrr^+^-containing electrolyte (Figure S4), confirming a permanent structural activation effect.

Comparison with 2D layered transition metal disulfides (e.g., TiS_2_) highlights the superior electrochemical performance of quasi-1D pseudolayered TiS_3_. Unlike TiS_2_, which require multiple cycles or low current density for activation, TiS_3_ achieves full activation after a single discharge at a relatively high current density of 100 mA g^–1^, offering higher reversible capacities (∼300 mA h g^–1^ vs ∼140 mA h g^–1^ for TiS_2_ at 100 mA g^–1^; Figure S5c) and elevated redox peak voltages (∼0.70 V/1.13 V vs ∼0.30 V/0.98 V for TiS_2_; Figures S4c and S5e). This advantage stems from its unique sulfur chemistry: the presence of reducible S_2_^2–^ moieties (as opposed to nonreducible S^2–^ in disulfides like TiS_2_, Figure S6) enables an S­(−I) + e^–^ ↔ S­(−II) redox process at higher voltages, providing additional, considerable capacity beyond Ti^4+^/Ti^3+^ redox. Furthermore, S_2_^2–^ species reduce electrostatic interactions, lower the energy barrier for BMPyrr^+^/magnesium ion intercalation, and enhance ionic mobility, collectively contributing to superior performance. Therefore, the unique sulfur chemistry provides TiS_3_ with distinct electrochemical advantages over TiS_2_ and other layered sulfides.

The TiS_3_ electrode also demonstrates excellent rate capability and high-current-density cycling stability in BMPyrr^+^-containing electrolyte. As shown in Figure 4d, the electrode delivers 310, 277, 242, 199, and 181 mA h g^–1^ at current densities of 100, 200, 400, 800, and 1000 mA g^–1^, respectively. Representative (dis)­charge curves at various current densities are provided in Figure 4e to emphasize the appearance of the respective profiles. When evaluating the long-term cycle performance at a high current density of 1000 mA g^–1^, the electrode demonstrates a good capacity of 168 mA h g^–1^ after 500 cycles (Figure 4g). Compared with recently reported chalcogenide cathodes for MIBs, our TiS_3_ electrode exhibits superior rate performance (Figure 4f),^32,49–57^ primarily due to its BMPyrr^+^-pillaring activated distinctive dual-redox chemistry and *in situ* generated nanosizing effects, as discussed below.

To elucidate the magnesium ion storage mechanism, *ex situ* and *in operando* techniques, including SEM-EDS, XPS, XAS, and PXRD, were employed. SEM images and EDS maps (Figures 5 and S7) reveal distinct microstructural transformations during cycling. At an early discharge to 0.6 V (1D0.6 V, ∼33 mA h g^–1^), the initially smooth-edged TiS_3_ particles develop cracks and lines along the sheet edges, indicative of the initiation of interlayer expansion along the *c*-axis triggered by BMPyrr^+^ intercalation (Figure 5a,d). EDS mapping reveals that intercalation at this stage primarily involves BMPyrr^+^ cations (Figure 5g). As the discharge progresses to 0.5 and 0.25 V (1D0.5 V, 1D0.25 V), thin submicron platelets form (Figure 5b,e and c,f), confirming significant delamination. EDS maps taken at this period confirm substantial cointercalation of BMPyrr^+^ and magnesium ions (Figure 5h,i). Fully discharged electrodes (1D0.01 V) show extensive exfoliation and formation of micronano thin platelets (Figure S7a,e). Upon charging to 2.0 V, exfoliated nanostructured platelets dominate, with similar morphological transformations observed during the second cycle (Figure S7b–d, f–h).

**Figure 5:**
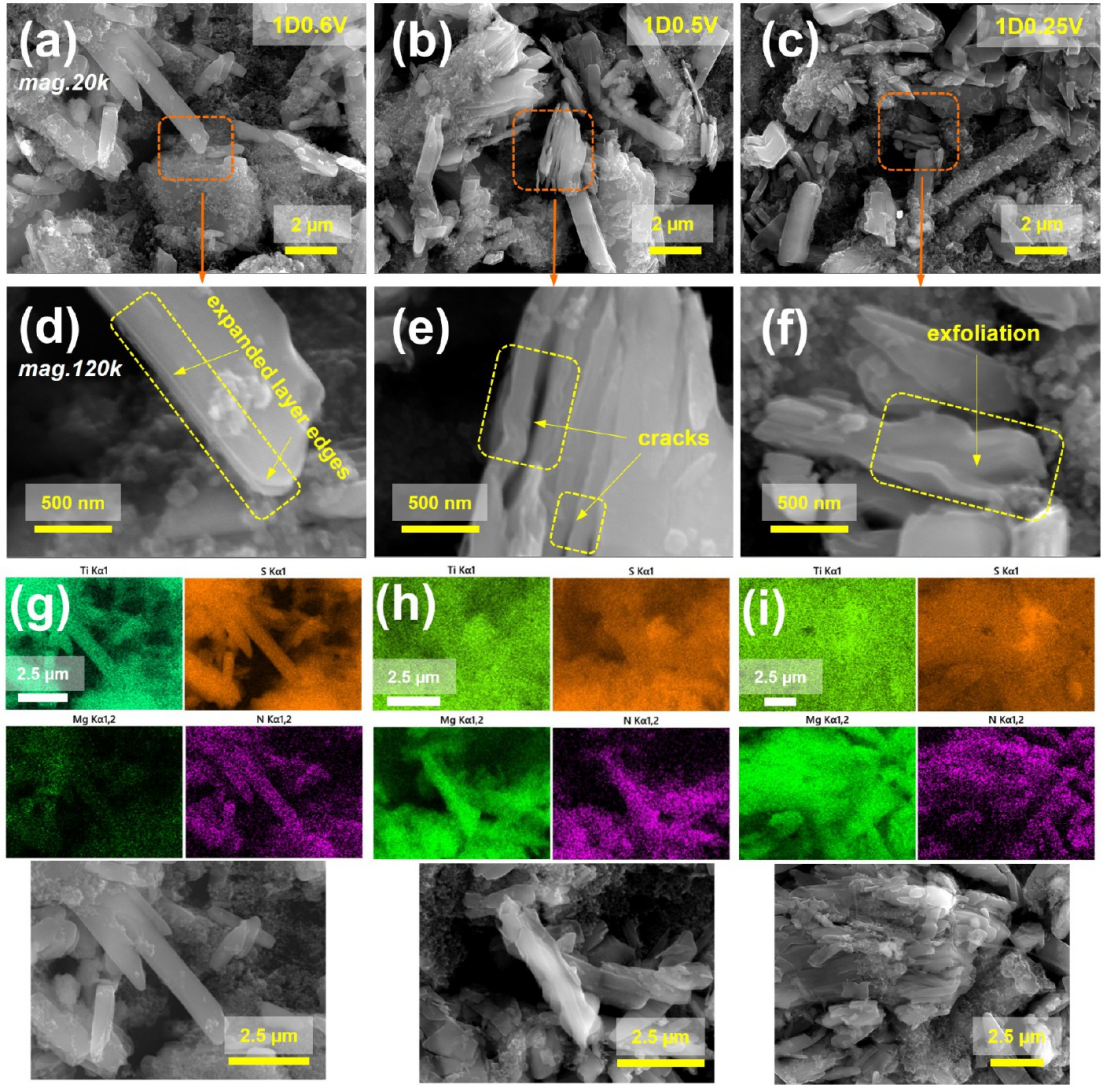
(a)–(f) SEM images (with magnifications of 20k and 120k) and (g)–(i) SEM/EDS elemental maps (Ti, S, Mg, and N) of the TiS_3_ electroactive particles at 1D0.6 V (a, d, g), 1D0.5 V (b, e, h), and 1D0.25 V (c, f, i) states of charge. Orange dashed lines and arrows in (a)–(c) highlight the regions that are magnified to (d)–(f).

EDS mapping across the cycling states confirms the uniform distribution of Ti, S, Mg, and N (Figure S7i–l). Quantitative EDS (Figure S8, Table S4) reveals Mg/Cl ratios ranging from 1.9 to 5.0, deviating from stoichiometric MgCl^+^ or Mg_2_Cl_3_^+^ in APC-based electrolytes, suggesting the coexistence of Mg^2+^ and Mg_*x*_Cl_*y*_^+^ as the primary intercalants, consistent with expanded VS_2_ electrodes.^16^ Both Mg/Ti and Cl/Ti ratios vary consistently with cycling, while Al/Ti ratios stabilize at ∼0.2, indicating electrolyte adsorption. Notably, a reduction of the S/Ti ratio from ∼3 to ∼2 during cycling points to partial decomposition of TiS_3_ to disulfide-like phases. While Arsentev *et al*. theoretically predicted Mg_0.5_TiS_3_ (186 mA h g^–1^) and more excessively intercalated TiS_3_ compounds to be unstable, proposing decomposition into titanium sulfides and MgS,^58^ our experiments suggest additional sulfur-rich byproducts. SEM-EDS investigations across the cathode, electrolyte, and Mg anode traced sulfur-rich deposits on the Ni current collector (Figure S10), implying that sulfur loss may involve sulfur-abundant compounds (e.g., carbon sulfides) formed through parasitic reactions with the electrolyte, apart from MgS as a possible minor product. Similar chalcogen loss has been reported or evidenced for other transition metal chalcogenides (e.g., VS_4_, TiS_2_, and TiSe_2_) in similar electrolytes.^28,29,59^ The titanium sulfide formed after the first discharge serves as the electroactive species in subsequent cycles, as magnesium ions are exclusively incorporated into its structure. The complex decomposition mechanism and the inactive sulfide byproduct(s), which cannot be conclusively deduced from the current data, would benefit from comprehensive investigations in future work. Assuming a three-electron transfer from Mg^2+^ and Mg_2_Cl_3_^+^ intercalation, the composition at the fully discharged state could approximate Mg_1.2_(Mg_2_Cl_3_)_0.6_TiS_3_ before decomposition. After decomposition, this compound transitions into Mg_1.15_(Mg_2_Cl_3_)_0.29_TiS_2_ as indicated by the EDS analysis, with hypothesized byproducts including Mg^2+^, Mg_2_Cl_3_^+^, and sulfur (or phenyl disulfide, Ph_2_S, *via* electrolyte interaction). An idealized, simplified overall decomposition pathway could be Mg_1.2_(Mg_2_Cl_3_)_0.6_TiS_3_ → Mg_1.15_(Mg_2_Cl_3_)_0.29_TiS_2_ + 0.05 Mg^2+^ + 0.31 Mg_2_Cl_3_^+^ + S (or Ph_2_S). Mg^2+^ and Mg_2_Cl_3_^+^ may return to the electrolyte, with Mg_2_Cl_3_^+^ potentially decomposing into Cl_2_ and Mg^2+^ (or MgCl^+^) during lattice degradation. Sulfur byproducts might react with nucleophilic APC electrolytes, forming organic sulfides (e.g., Ph_2_S), which could undergo parasitic reactions, depositing solid organic sulfides onto the current collector.^60^ This decomposition hypothesis accounts for both the observed loss of sulfur and the reduction in magnesium content within the TiS_3_ electrode (equivalent to 0.41 *e* ^–^/TiS_3_) as indicated by the EDS analysis. To verify these assumptions, advanced characterization techniques are proposed. *In operando* Raman spectroscopy could identify sulfur-containing bonds in liquid or solid states,^61^ nuclear magnetic resonance (NMR) spectroscopy could probe solid organic sulfide byproducts,^62^ and online electrochemical mass spectrometry (OEMS) could detect potential gaseous species (e.g., sulfur oxides and Cl_2_) generated during decomposition.

The presence of N, Mg, Cl, and valence states of Ti and S in the TiS_3_ electrode materials were investigated using XPS during cycling. High-resolution N 1s spectra (Figure 6a) at discharged and charged states reveal two categories of N signals: a peak at 402.6 eV corresponding to BMPyrr^+^ (quaternary nitrogen cation),^63^ and a peak at 400.3 eV associated with pyridine radical (N^+**·**^),^64^ likely due to trace water-induced inevitable side reactions during electrode washing and transfer. Furthermore, depth profiling using Ar^+^ etching (Figure S11) reveals a consistent nitrogen signal extending approximately 510 nm into electrodes at both charged and discharged states. These results confirm the penetration of BMPyrr^+^ cations into the bulk electrode and their retention within the electrode throughout the discharge–charge cycle. The Mg 2p spectra (Figure 6b) show a peak at 50.7 eV, while the Cl 2p spectra (Figure 6c) display two overlapping peaks in the range of 196–202 eV. Notably, the intensities of these peaks increase upon discharge and decrease with charge, confirming Mg^2+^/Mg_*x*_Cl_*y*_^+^ (de)­intercalation. Additionally, depth-dependent Cl 2p spectra (Figure S12) further corroborate the presence of Mg_*x*_Cl_*y*_^+^ intercalants within the electrode. The measurement of the Ti 2p level was automatically focused on Ti 2p_3/2_ region; therefore, only the Ti 2p_3/2_ peaks of the various electrodes are presented, each exhibiting several deconvoluted components (Figure 6d). Peaks at 458.8 and 457.2 eV are attributed to TiO_2_ and TiS_*x*_O_*y*_, respectively, possibly formed from (partial) oxidation during electrode washing and transfer.^43^ The two peaks at lower binding energies of 456.0 and 455.5 eV correspond to the 4+ and 3+ Ti states in the titanium sulfide active materials, respectively. The presence and absence of Ti^3+^ at discharged and charged states suggest a reversible Ti^4+^-to-Ti^3+^ redox process. In parallel, S 2p spectra (Figure 6e) show the weakening of S_2_^2–^ peaks (162.5/163.7 eV) and strengthening of S^2–^ peaks (161.1/162.2 eV) upon discharge, which partially reverses during charge, confirming an anionic S_2_^2–^/S^2–^ redox component. The increased proportion of S^2–^ peaks after prolonged cycling is likely related to the previously discussed surface decomposition, which generates possible S^2–^-containing phases (e.g., TiS_2_-like or MgS_*x*_ species). In addition, the broadened S 2p envelope after cycling reflects the interference of poorly conductive surface films and the loss of long-range order at the electrode surface.

**Figure 6:**
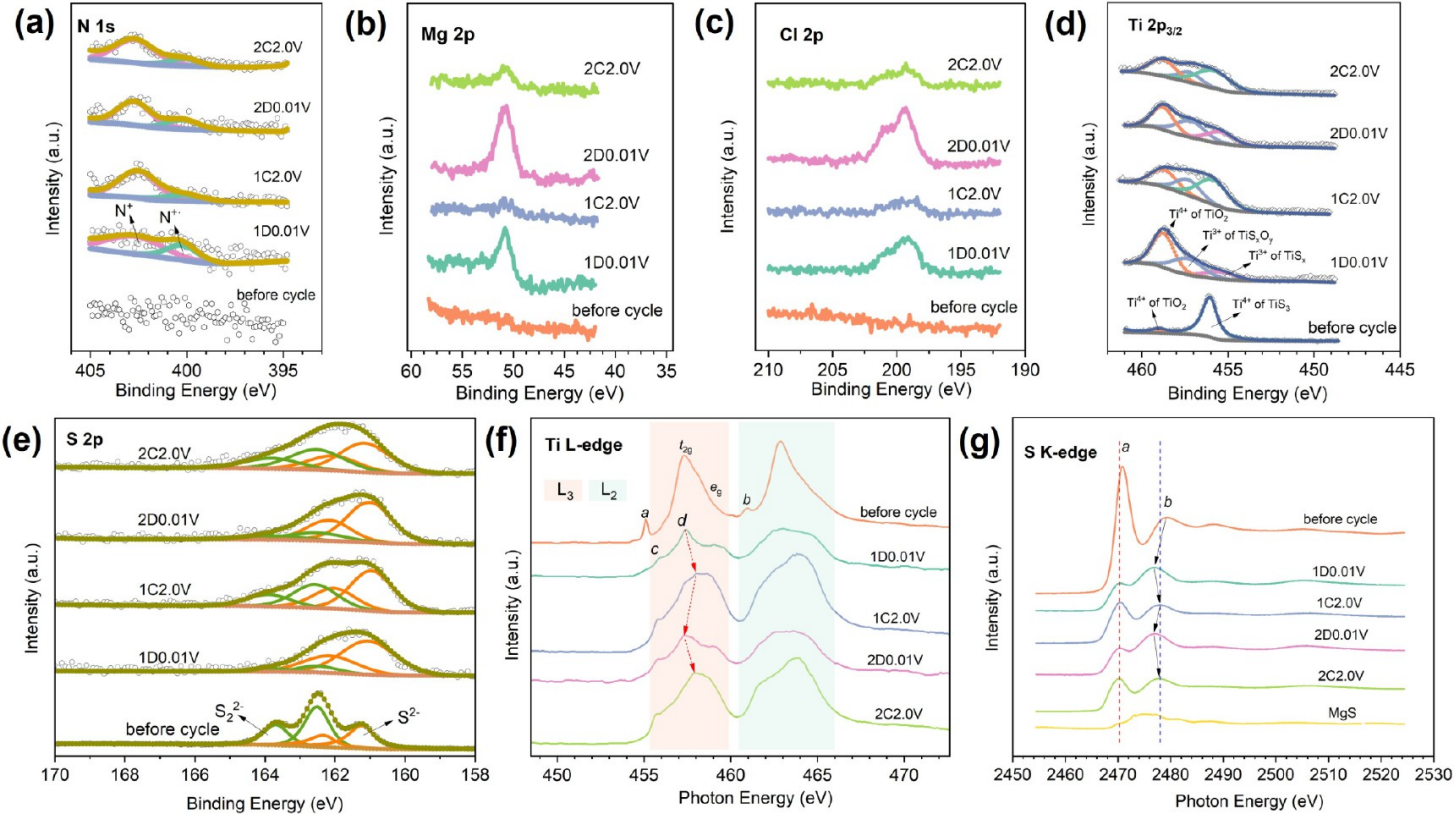
High-resolution XPS spectra of (a) N 1s, (b) Mg 2p, (c) Cl 2p, (d) Ti 2p_3/2_, and (e) S 2p transitions; XAS spectra of the (f) Ti L-edge and (g) S K-edge in the TiS_3_ electrodes at various discharge and charge states. MgS standard data were also collected for reference.

XAS was further employed to corroborate the cationic and anionic redox processes during cycling. As shown in Figure 6f, the Ti L-edge spectrum of pristine TiS_3_ powder, largely determined by a transition probability from 3d^0^ to 2p^5^3d^1^, consists of the L_3_ (454.2–460.0 eV) and L_2_ (460.5–467.0 eV) edges, corresponding to Ti 2p_3/2_ and Ti 2p_1/2_ excitations, respectively.^42^ Both edges exhibit greatly overlapped *t*_*2g*_ and *e*_*g*_ orbitals split by the crystal field. These orbitals are directly involved in 2p to 3d transitions, and their energy levels are affected by changes in oxidation state, especially for *e*_*g*_ orbitals that direct overlap with ligand orbitals (e.g., S p-orbitals in sulfides). Due to the shorter lifetime of the 2p_1/2_ core hole and broadening, low resolution of the L_2_-edge peaks,^65^ analysis focuses on the better-resolved L_3_-edge peaks. The small pre-edge features, *a* and *b*, are associated with multiplet core hole–3d electron interactions.^66^ After discharge and charge cycles, the absorption edges of the Ti centers exhibit noticeable differences compared to those of the pristine TiS_3_ powder, attributable to changes in the oxidation states and coordination environment of Ti, as the material transitions from a trisulfide to a disulfide structure. Indeed, the XAS peak profiles of the transformed titanium sulfide after cycling resemble those of TiS_2_, TiSe_2_, and TiTe_2_,^67^ indicating possible similar Ti coordination symmetry. At the charged states, the *c* and *d* peaks at 455.8 and 458.0 eV are comparable to ∼455.9 and 458.0 eV for Ti^4+^ in TiS_2_ and ∼455.9 and 457.9 eV for Ti^4+^ in Li_1.13_Ti_0.57_Fe_0.3_S_2_.^67,68^ At the discharged states, these peaks are positioned at 455.8 and 457.4 eV, comparable to ∼455.8 and 457.3 eV for Ti^3+^ in Ni_0.46_TiSe_2_.^69^ The above shifts of the L_3_ edge main peak by ∼0.6 eV during cycling, along with the previous XPS results, corroborate the reversible Ti^4+^-to-Ti^3+^ redox. The S K-edge spectra (Figure 6g) reveal two main regions: a pre-edge peak *a* at 2470.9 eV, attributed to the unoccupied S 3p/Ti 3d hybridized states, and a broad peak *b* at 2479.2 eV, representing the transitions to higher states, e.g., S 1s to S 3p/Ti 4s, 3p.^68^ After cycling, peaks *a* and *b* are located at energies of 2470.3 eV and a range from 2476.9 to 2478.1 eV, respectively, compared to those of TiS_3_ (2470.9 and 2479.2 eV) and MgS (an energy range from 2469.4 to 2483.4 eV). These results indicate adjustments in the electronic structure of the electrode material upon cycling, which merely involve MgS. The increase or decrease in the intensity of peak *a* during charge and discharge cycles relates to periodic changes in the density of unoccupied S 3p/Ti 3d hybridized states just above the Fermi level. This suggests electrons are removed from or added to the S 3p/Ti 3d orbitals as magnesium ions are deintercalated or intercalated, indicating reversible oxidation/reduction of sulfur (S^2–^ ↔ S_2_^2–^) and titanium (consistent with the Ti L_3_-edge changes). Concurrently, peak *b* shifts (∼1.2 eV) during cycling reflect the variations in the effective nuclear charge *Z*_eff_, an indicator for redox reaction at the sulfur “ligands”.^68,70^ This suggests the removal or injection of electrons in sulfur ligands of titanium sulfide during charge (shift to higher energy) and discharge (shift to lower energy), confirming redox activity at sulfur sites, consistent with the literature.^68^ These results corroborate a dual cationic (Ti^4+^ ↔ Ti^3+^) and anionic (S_2_^2–^ ↔ S^2–^) redox process during cycling, consistent with XPS findings.

In-house *in operando* lab PXRD techniques were further employed to probe the structural evolution of the TiS_3_ electrode during the initial two (dis)­charge cycles. The contour graph of the *in operando* patterns and corresponding galvanostatic (dis)­charge curves are portrayed in Figure 7a. During the first discharge to 0.47 V, a set of new peaks for an expanded TiS_3_-type *Phase 1* Mg_*k*_(Mg_*x*_Cl_*y*_)_*m*_(BMPyrr)_*n*_TiS_3–*z*_ (highlighted by pink band) gradually emerge at 2*θ* values of ∼6.4°, 12.7°, 19.4°, 26.0°, 32.8°, 37.2°, 39.4°, 46.1°, and 46.6° (Figure 7a and Figure S13a–c). Among them, the peak at 2*θ* ≈ 6.4° is assigned to the expanded (001) interlayer plane with a *d* spacing of ∼1.39 nm, while the peaks at 2*θ ≈* 12.7°, 19.4°, 26.0°, 32.8°, 39.4°, and 46.1°, with *d* spacings being integer factors *l* of 1.39 nm, are indexed as (00*l*) reflections of the expanded *Phase 1*. Rietveld refinement against *ex situ* PXRD data at 1D0.54 V (Figure S13a,b and Table S5) confirms a *c*-axis expansion and significant volume increase (*c* = 13.813­(3) Å, 231.1(4) Å^3^) compared to 147.24(6) Å^3^ for pristine TiS_3_. This expanded interlayer, with a vdW gap of 0.84 nm (1.39 nm – 0.55 nm = 0.84 nm; thickness of TiS_3_ pseudolayer ≈0.55 nm), cannot be explained by Mg^2+^ or Mg_*x*_Cl_*y*_^+^ insertion (length ≤ 0.31 nm), as supported by the absence of observable peak shifts in the TiS_3_ PXRD pattern after discharge in APC (32 mA h g^–1^, Figure S13d). In contrast, such expansion aligns with the larger BMPyrr^+^ cations (∼0.7–0.8 nm) intercalating perpendicularly to the pseudolayers, acting as structural pillars similar to alkylamine-intercalated TiS_2_.^71^ Upon further discharge to 0.01 V, another new peak at 35.5° arises and shifts slightly to progressively lower 2*θ* positions, corresponding to a *d* spacing that is not an integral factor of 1.39 nm, thereby likely associated with lattice expansion along at least one other direction, such as the weak interchain along the *a-*axis. Additionally, the PXRD pattern of the pristine TiS_3_ electrode immersed in the APC-BMPyrrCl electrolyte (Figure S13e) shows no discernible shifts in peak positions, suggesting that BMPyrr^+^ intercalation does not occur spontaneously and requires an electrochemically driven process.

**Figure 7:**
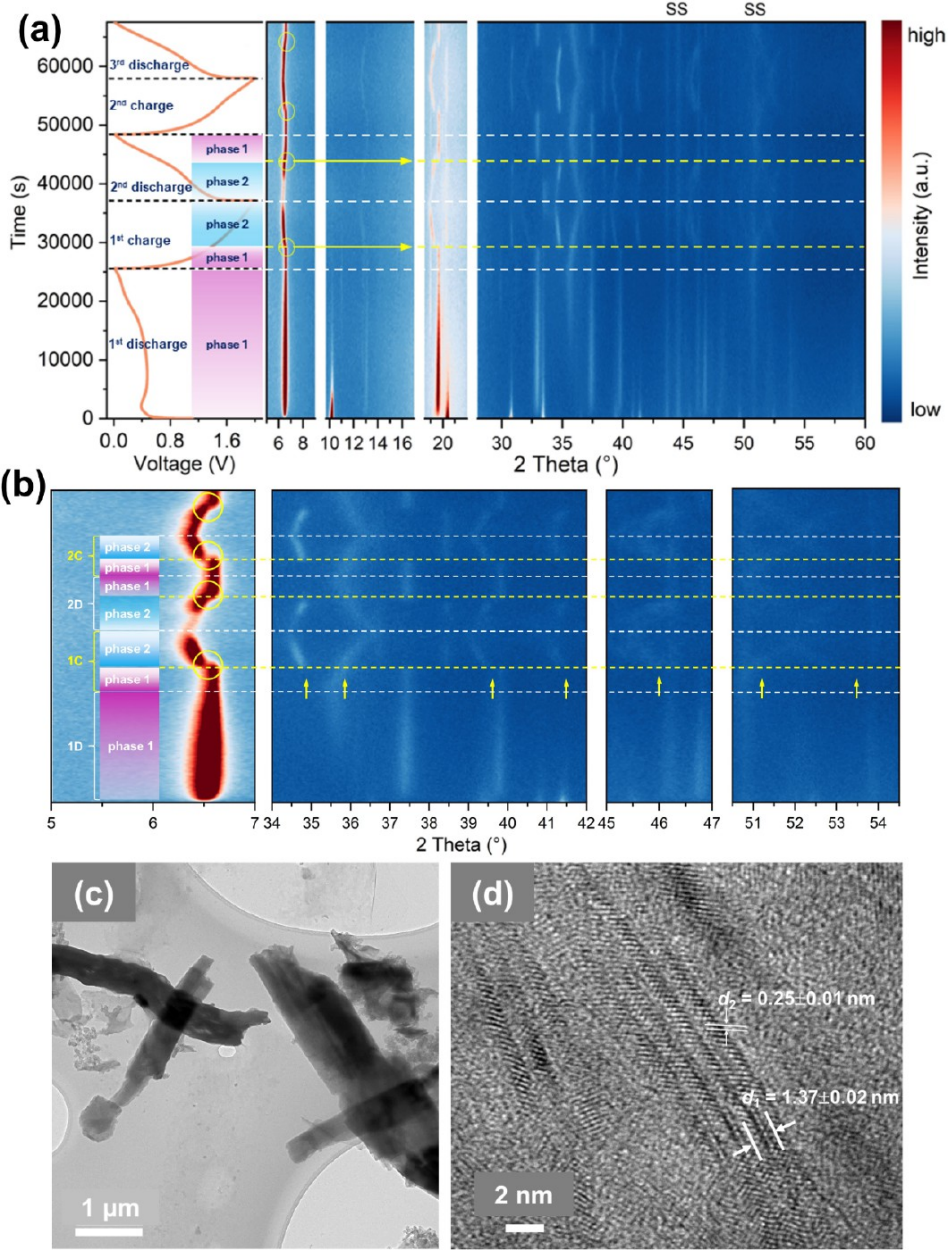
(a) Contour graph of *in operando* PXRD patterns (right) and corresponding galvanostatic (dis)­charge curves (left) acquired at a current density of 100 mA g^–1^. The graduated light purple bands and light blue bands in the charge/discharge plots indicate the progressive (de)­intercalation in *Phases 1 and 2*. Reflections for stainless steel are denoted as SS. White and yellow dashed lines represent the discharge/charge and phase transition boundaries, respectively. (b) Enlarged *in operando* PXRD plot regions taken from (a). Yellow circles, arrows, and dashed lines highlight the phase transition. White dotted lines indicate the boundaries between the discharge and charge steps. (c) TEM image and (d) HRTEM image of the 1^st^ discharged TiS_3_ electrode.

Upon the first charging to 1.5 V, the PXRD patterns show reversible changes opposite to those observed during the initial discharge. As charging proceeds, a phase transition to *Phase 2* emerges (blue bands), as seen from the discontinuous peak shifts (yellow circles and arrows). Further charging to 2.0 V slightly increases the interlayer distance to *∼*1.40 nm, which indicates a change in the Coulombic interactions between intercalated magnesium ions and S_2_^2–^/S^2–^ sublattice, analogous to intercalation of Li^+^ (high charge density, small size), Na^+^ and K^+^ ions (low charge density, large size) into TiS_2_.^72–74^ At the end of the first charge, the lattice structure remains expanded, with BMPyrr^+^ serving as a stabilizing pillar. The minor structural changes between expanded *Phase 1* and *Phase 2* persist in subsequent cycles, enabling rapid magnesium ion diffusion and improved capacity.

*Ex situ* capillary PXRD patterns (transmission geometry) in Figure S14 further corroborate the *in operando* results, showing the disappearance of the original TiS_3_ phase and formation of new phases with lattice expansion and contraction linked to the discharge and charge states. Reduced incidence of preferred orientation in capillary vs flat plate geometry clarifies certain features. For example, the peak at *∼*35.6°, which emerges at the 1D0.45 V state in the *in operando* patterns, actually grows from the 1D0.54 V state in the *ex situ* patterns. The *ex situ* TEM image (Figure 7c) reveals the micro-to-nanosized platelet morphology of the first discharged sample, with exfoliated nanosheets prominently decorating the larger particles. In the corresponding HRTEM image (Figure 7d), lattice fringes with spacings of *d*_1_ = 1.37 ± 0.02 nm and *d*_2_ = 0.25 ± 0.01 nm are observed, corroborating the interlayer and potential interchain expansion identified in the *in operando* PXRD analysis, respectively.

Based on the combined EDS, XPS, XAS, PXRD, and TEM studies during (dis)­charge cycles, a possible interpretation of the structural evolution in the TiS_3_ electrode is graphically demonstrated in Figure 8. At the early stage of the discharge (1D0.6 V), bulk BMPyrr^+^ cations intercalate into the interlayers, forming interlayer-expanded structure with interlayer gaps increasing from *∼*0.32 to 0.82 nm. When fully discharged, a substantial number of Mg^2+^/Mg_*x*_Cl_*y*_^+^ and some BMPyrr^+^ ions cointercalate, slightly reducing the interlayer spacing (Δ*d* ≈ 0.03 nm) due to increased Coulombic attractions. Upon the first charge (2.0 V), most magnesium ions are extracted, leaving BMPyrr^+^-pillared frameworks that preserve expanded channels for subsequent cycles.

**Figure 8:**
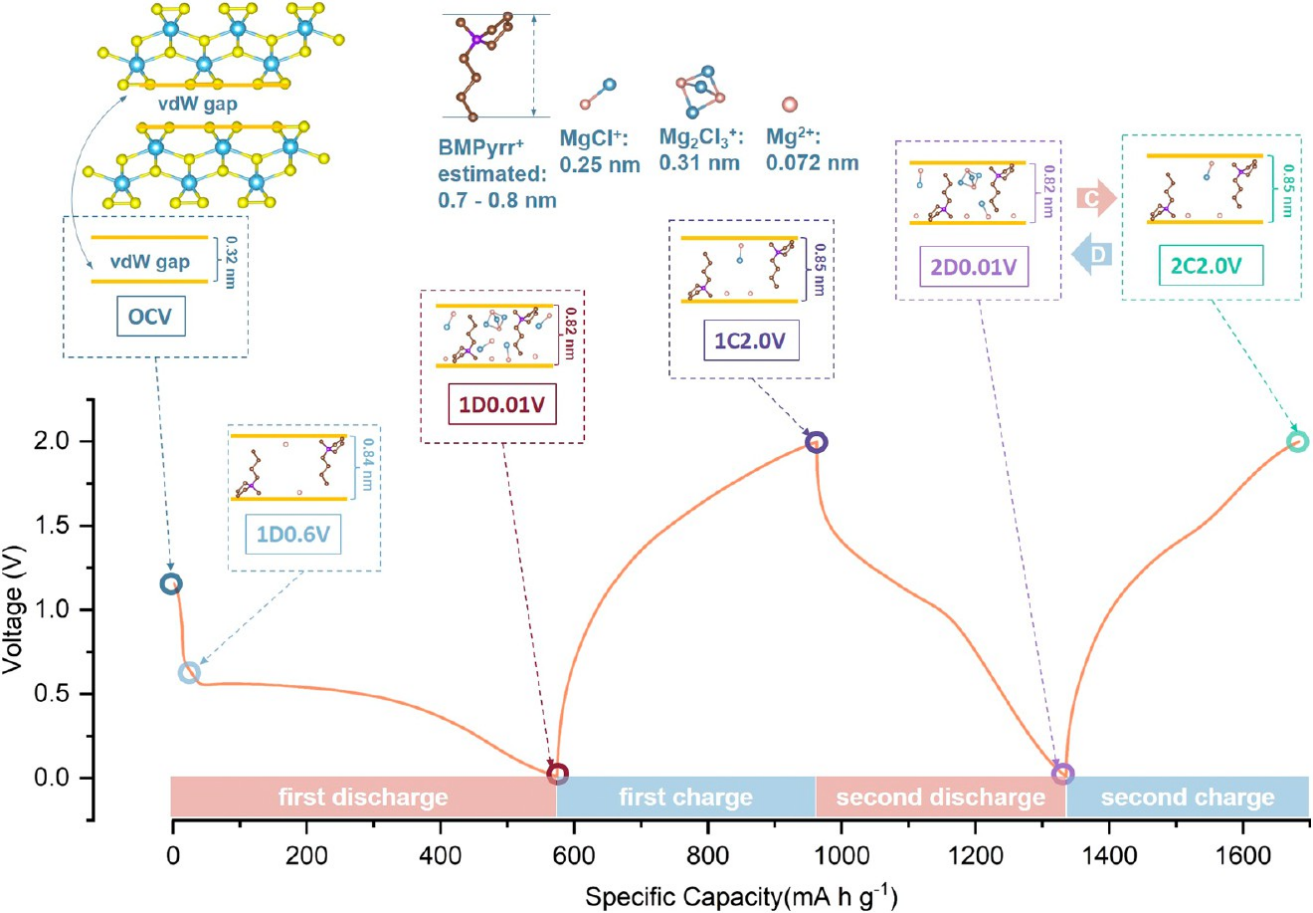
Proposed schematic of a simplified BMPyrr^+^ and magnesium ion intercalation/deintercalation processes in the TiS_3_ electrode and the resulting structural evolution during (dis)­charge cycles. Note that this is a proposed mechanistic illustration and does not represent real crystal structures in the electrodes.

The charge storage mechanism, ion transport kinetics, and interfacial properties of TiS_3_ electrodes were comprehensively investigated using CV, GITT, and EIS techniques. CV curves at scan rates of 0.2, 0.4, 0.6, and 0.8 mV s^–1^ were analyzed using Equations S1 and S2 to determine the *b* constant, which distinguishes between diffusion-controlled (*b* ≈ 0.5) and pseudo capacitive (*b* ≈ 1) processes. The *b*-values for the main cathodic and anodic peaks are 0.856 ± 0.007 and 0.832 ± 0.015, respectively (Figure 9b), indicating a mixed diffusion/pseudo capacitive storage mechanism. To quantify these contributions further, current responses at various scan rates were deconvoluted using Equation S3. As shown in Figure 9c, the pseudo capacitive contributions increase with scan rate, reaching 58%, 65%, 69%, and 74% at 0.2, 0.4, 0.6, and 0.8 mV s^–1^, respectively. This shift toward surface-controlled storage at higher scan rates is attributed to increased surface areas through *in situ* particle downsizing and defect generation during the first discharge, which expose abundant active sites and facilitate rapid charge transfer. The dominance of pseudo capacitive behavior at higher scan rates rationalizes the high rate performance of TiS_3_ compared with other MIB cathodes (Figure 4f).

**Figure 9:**
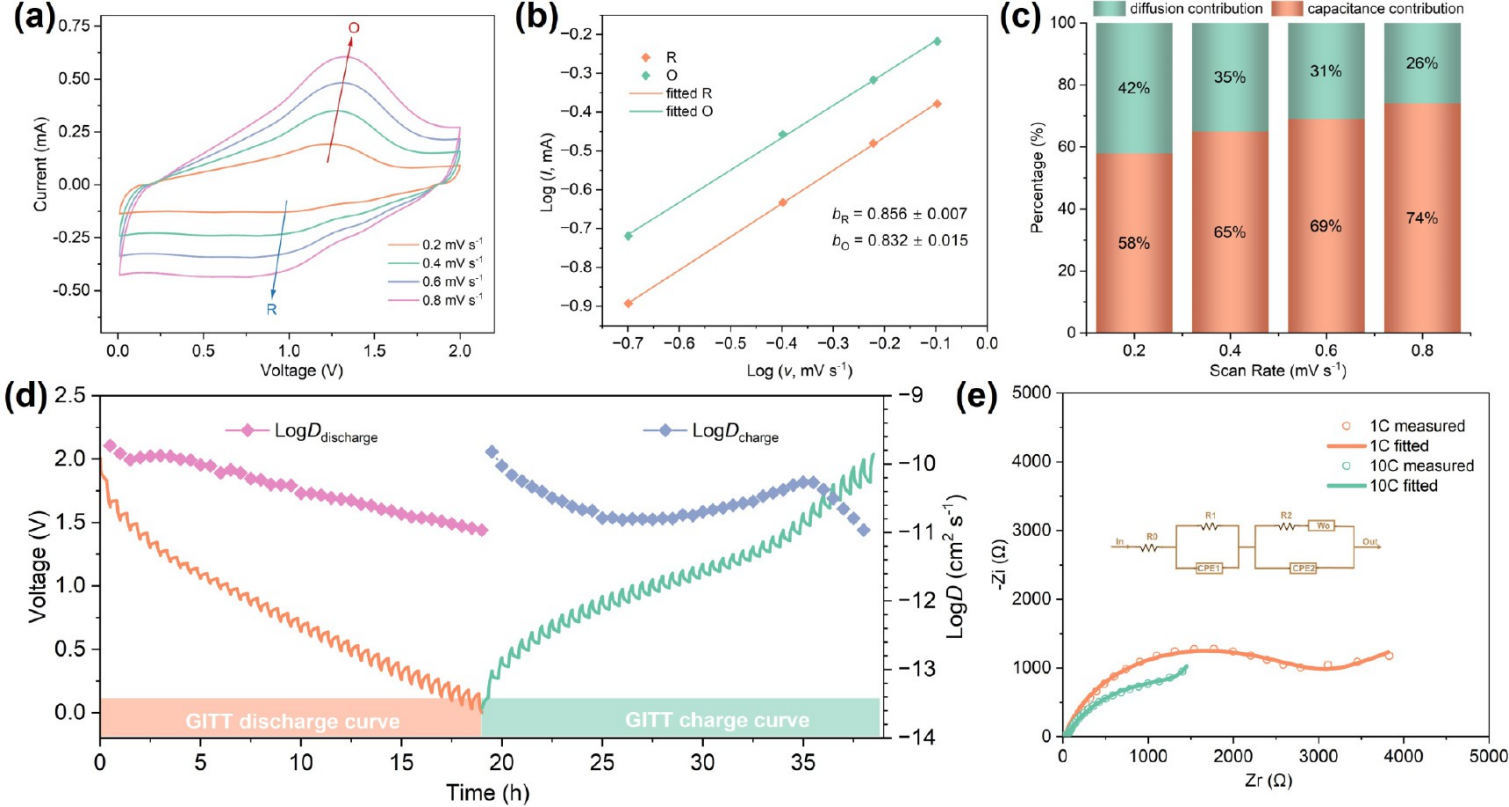
Electrochemical charge storage mechanism, ion diffusion kinetics, and interphase properties of the TiS_3_ electrodes in Mg|APC-BMPyrrCl|TiS_3_ cells: (a) CV curves of the TiS_3_ electrode obtained at different scan rates of 0.2 mV s^–1^ (orange), 0.4 mV s^–1^ (cyan), 0.6 mV s^–1^ (violet), and 0.8 mV s^–1^ (pink), respectively. (b) Plots of the measured and fitted log oxidation (cyan) and log reduction (orange) peak currents against the log of scan rates *v*, where the solid lines represent the linear fits (adjusted *R* ^2^ values, *R*_O_^2^ = 0.9991, *R*_R_^2^ = 0.9998). (c) Histogram of the capacitive and diffusive proportions at different scan rates. (d) Plots of the GITT discharge–charge curves (orange and cyan lines and shadowed bands) at a current density of 50 mA g^–1^ and of the corresponding discharge and charge diffusivities (pink and violet dotted lines). (e) Nyquist plots (hollow circles) and corresponding fit curves (solid lines) of the electrode after the first (orange) and tenth (turquoise/cyan) charges, respectively. The equivalent circuit is inset in the graph.

Subsequently, GITT profiles (Figure 9d) were analyzed to determine the diffusion coefficients of Mg^2+^/Mg_*x*_Cl_*y*_^+^ in the TiS_3_ electrode using Equation S4. During discharge and charge, *D* values range from 1.09 × 10^–11^ cm^2^ s^–1^ to 1.87 × 10^–10^ cm^2^ s^–1^, and from 1.09 × 10^–11^ cm^2^ s^–1^ to 1.52 × 10^–10^ cm^2^ s^–1^, respectively, with an average *D*_discharge_ of 6 × 10^–11^ cm^2^ s^–1^, comparable to values reported for other expanded chalcogenides (Table S6). EIS experiments were employed to assess charge transfer resistance and interfacial characteristics over cycling. The Nyquist plots (Figures S16 and 9e) were fitted (using Aftermath software) employing an equivalent circuit comprising ohmic resistance (R_0_), SEI resistance (R_1_ + CPE_1_), charge-transfer resistance (R_2_ + CPE_2_), and Warburg impedance (W_0_),^75,76^ with specific fitting parameters shown in Table S7. Initially, R_1_ drops considerably from *∼*2152 Ω to *ca.* 31 Ω after the first charge and stabilizes over ten cycles. This suggests an “activation” process consisting of an efficient breakage of the passivation/oxidation layer and the establishment of a new conductive SEI for interfacial mass transport. Similarly, R_2_ decreases from ∼13444 Ω to 2018 Ω after 10 cycles, and W_0_ decreases from 349 Ω s^–0.5^ to 2159 Ω s^–0.5^, indicating that magnesium ion transport and charge transfer become significantly more efficient after structural activation. The above EIS observations strongly suggest that the pillaring effect of BMPyrr^+^ and particle size reduction facilitate charge transfer, shorten the magnesium ion transport pathways, and result in more favorable diffusion on/within the electrode material. Similar improvements have been observed in organic-species-intercalated chalcogenides such as 2-ethylhexylamine-*pre*-intercalated VS_2_ and BMPyrr^+^-*in situ*-intercalated vanadium molybdenum disulfide and TiS_2_.^16,28,32^

## 4 Conclusions

In summary, this work demonstrates that interlayer expansion, achieved *via* the electrochemical intercalation of BMPyrr^+^ cations, is a powerful strategy to enhance magnesium ion storage in a structurally unique pseudolayered TiS_3_ cathode featuring redox-active Ti^4+^ and S_2_^2–^ species. The BMPyrr^+^-pillared TiS_3_ electrode delivers high reversible capacities of up to 300 mA h g^–1^, superior rate capability, and long-term cycling stability in APC-BMPyrrCl electrolyte, in stark contrast to the negligible capacity observed in the *unmodified* system. Mechanistic investigations reveal that BMPyrr^+^ intercalation expands the vdW gap from ∼0.32 nm to ∼0.82 nm, reducing electrostatic barriers, accelerating magnesium ion transport, and activating dual cationic (Ti^4+^/Ti^3+^) and anionic (S_2_^2–^/S^2–^) redox processes. Together with nanosizing-induced pseudocapacitance, these structural and electronic modifications synergistically underpin the enhanced electrochemical performance. Furthermore, structural and cycling performance analyses confirm that the intercalated BMPyrr^+^ species are retained within the TiS_3_ host after activation, sustaining the expanded interpseudo-layer channels for fast magnesium ion transport. This study establishes pseudolayered TiS_3_ as a versatile platform that integrates structural flexibility with dual-redox chemistry, highlighting the broader potential of interlayer engineering for next-generation magnesium ion and multivalent batteries. Looking ahead, unraveling the decomposition pathways and sulfur conversion reactions during deep magnesiation will be critical to achieving reversible capacities potentially exceeding 500 mA h g^–1^ and guiding the design of high-energy, long-life multivalent cathodes.

## Supplementary Material



## Notes

Experimental section, PXRD patterns, Rietveld refinement results, schematic illustrations of the crystal structures, SEM images, EDS spectra, TG-DSC curves, CV curves, (dis)­charge curves, histogram graph of atomic ratios, peak area of N 1s region, one pair of current pulse and relaxation data, Nyquist plots, XPS spectra, EIS spectrum, crystallographic data, atomic positional parameters, as-detected atomic composition, comparisons of the average diffusion coefficients, and detailed values of parameters (PDF)
