# Supplementary material for: Electrochemical Interlayer Expansion and Dual Redox Activation for Fast Mg-Ion Transport and High Capacity in Quasi-1D TiS 3
Source: ACS Sustain Chem Eng. 2026 Jan 9;14(3):1297–311. doi: 10.1021/acssuschemeng.5c09578 (PMC12849046; doi:10.1021/acssuschemeng.5c09578)
Supplement: Supplementary file 1 [file sc5c09578_si_001.pdf]

Supporting Information

Electrochemical Interlayer Expansion and Dual Redox Activation for Fast Mg-Ion Transport and High Capacity in Quasi-1D TiS_3

*Pengcheng Jing,[a1] Atsushi Inoishi,[b] Chengcheng Zhao,[a2] Eiichi Kobayashi,[c] Yisong Han,[d] and Duncan H. Gregory[a3]**

* Corresponding author

[a1] WestCHEM, School of Chemistry, Joseph Black Building, University of Glasgow, Glasgow, UK, G12 8QQ. E-mail: p.jing.1@research.gla.ac.uk

[b] Institute for Materials Chemistry and Engineering, Kyushu University, Kasuga-koen 6-1, Kasuga, Fukuoka, Japan, 816-8580. E-mail: inoishi@cm.kyushu-u.ac.jp

[a2] WestCHEM, School of Chemistry, Joseph Black Building, University of Glasgow, Glasgow, UK, G12 8QQ. E-mail: 2792248z@student.gla.ac.uk

[c] Kyushu Synchrotron Light Research Center, 8-7 Yayoigaoka, Tosu, Saga, Japan, 841-0005. E-mail: kobayashi@saga-ls.jp

[d] Department of Physics, University of Warwick, Coventry, UK, CV4 7AL. E-mail: yisong.han@warwick.ac.uk

[a3] WestCHEM, School of Chemistry, Joseph Black Building, University of Glasgow, Glasgow, UK, G12 8QQ. E-mail: Duncan.Gregory@glasgow.ac.uk. Tel: +44-141-330-8128

Total number of pages: 20

Total number of figures: 16

Total number of tables: 7

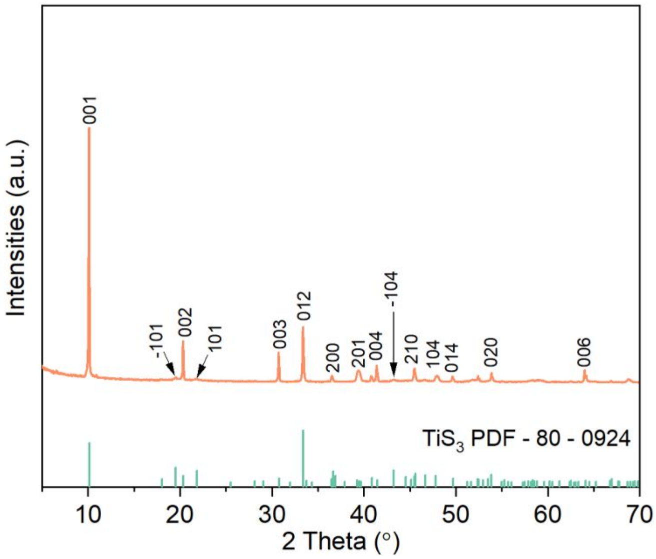

Figure S1. PXRD pattern (flat plat, reflection mode, Bragg-Brentano geometry) of the as-made TiS_3 powder and the corresponding standard pattern of a monoclinic TiS_3 phase with a registration num. of PDF - 80 - 0924.

Table S1. Crystallographic data obtained from Rietveld refinement of the as-made TiS_3 as compared to the published data.[1]

Chemical Formula	Synthesised TiS_3	Published TiS_3
Crystal System	Monoclinic	Monoclinic
Space Group	$P2_1/m$	$P2_1/m$
Lattice Parameter	a = 4.9702(3) Å, b = 3.4020(1) Å, c = 8.8046(6) Å, β = 97.64°(1)	a = 4.958(2) Å, b = 3.4006(11) Å, c = 8.778(4) Å, β = 97.32°(4)
No. of Points	5706	-
N – P + C	5694	-
Formula Weight / g mol^{-1}	143.9	143.9
Calculated Density / g cm^{-3}	3.242	3.260
Volume / Å^3	147.55(1)	146.79
R_{wp}	5.41%	
R_p	2.60%	
χ^2	1.67	

Table S2. Atomic positional parameters (z refined) and isotropic displacement parameters (B_{iso}) used in the Rietveld refinement of as-prepared TiS_3.

Atom	Label	x	y	z	Occ.	B_{iso} (Å^2)
Ti	Ti0	0.7187	0.2500	0.3544(4)	1	0.5

S	S0	0.1170	0.2500	0.8248(8)	1	0.9
S	S1	0.7605	0.7500	0.5658(5)	1	0.6
S	S2	0.4808	0.7500	0.1754(7)	1	0.9

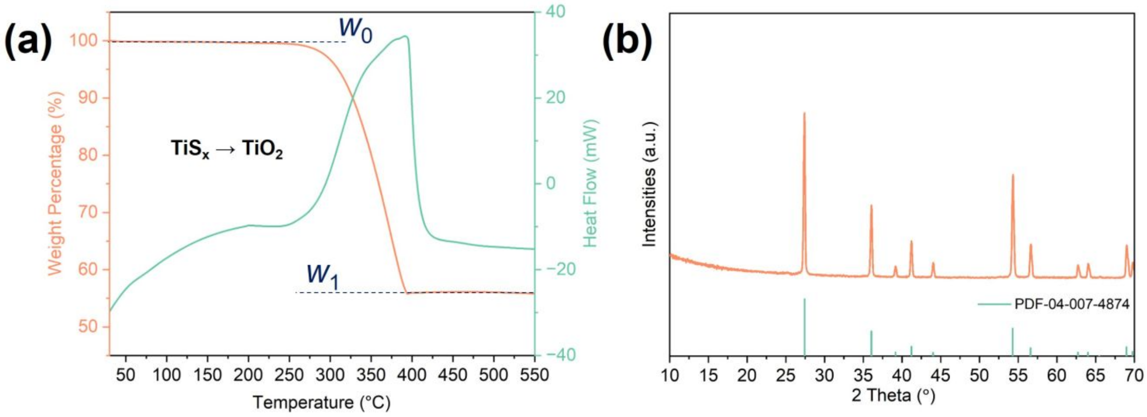

Figure S2. TG-DSC curves of the as-prepared TiS_3 powder and the corresponding PXRD pattern of the measurement product (single phase TiO_2). Calculation details are provided below.

$TiS_x + (xn/2)O_2 = TiO_2 + xSO_n$, $\Delta(TiS_x - TiO_2)$, $n = 2$ and/or 3

M_{TiSx} M_{TiO2} $\Delta M(M_{TiSx} - M_{TiO2})$

w_0 w_1 $\Delta\ w_{(w0\ -\ w1)}$

Therefore:

$$\frac{\Delta M_{(TiS_x - M_{TiO_2})}}{\Delta w_{(w_0 - w_1)}} = \frac{M_{TiO_2}}{w_1}, \frac{32(x-1)}{\Delta w_{(w_0 - w_1)}} = \frac{M_{TiO_2}}{w_1}$$

$$x = \frac{M_{TiO_2} \times \Delta w_{(w_0 - w_1)}}{32 w_1} + 1$$

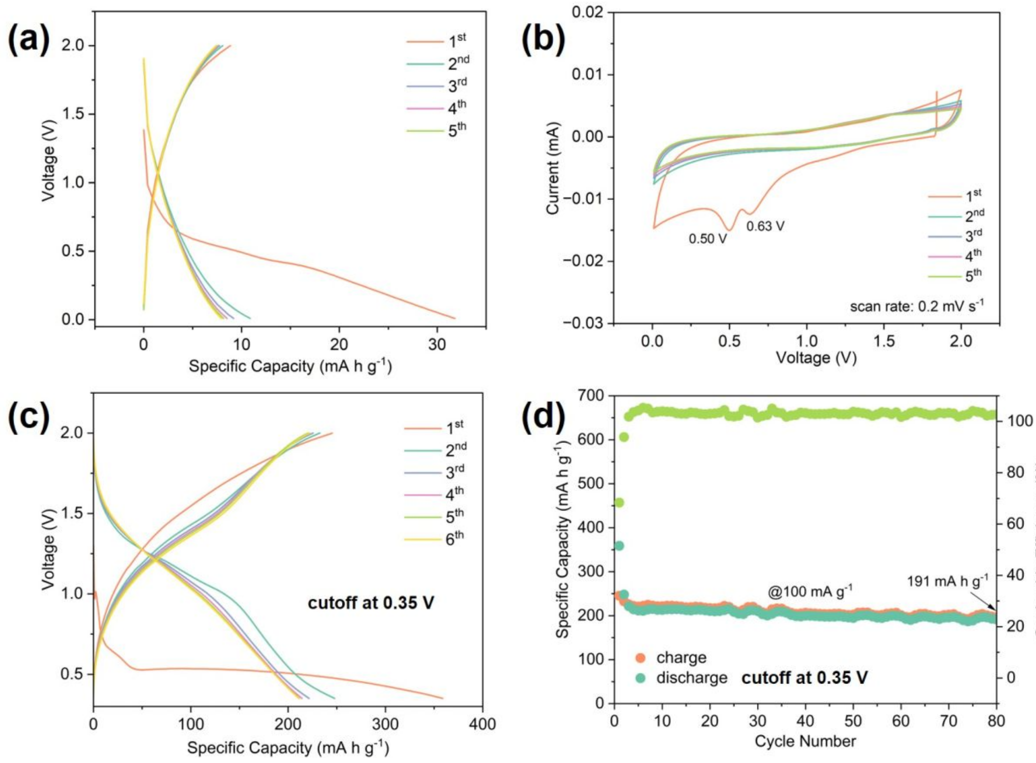

Figure S3. Electrochemical measurements of the TiS_3 electrode with pure APC electrolyte: (a) initial five cycles of galvanostatic (dis)charge curves at a current density of 100 mA g^{-1} and (b) CV curves at a scan rate of 0.2 mV s^{-1}. Electrochemical performance of the TiS_3 electrode with APC-BMPyrrCl electrolyte (cutoff at 0.35V): (c) initial six cycles of galvanostatic (dis)charge curves and (d) cycling performance at a current density of 100 mA g^{-1}.

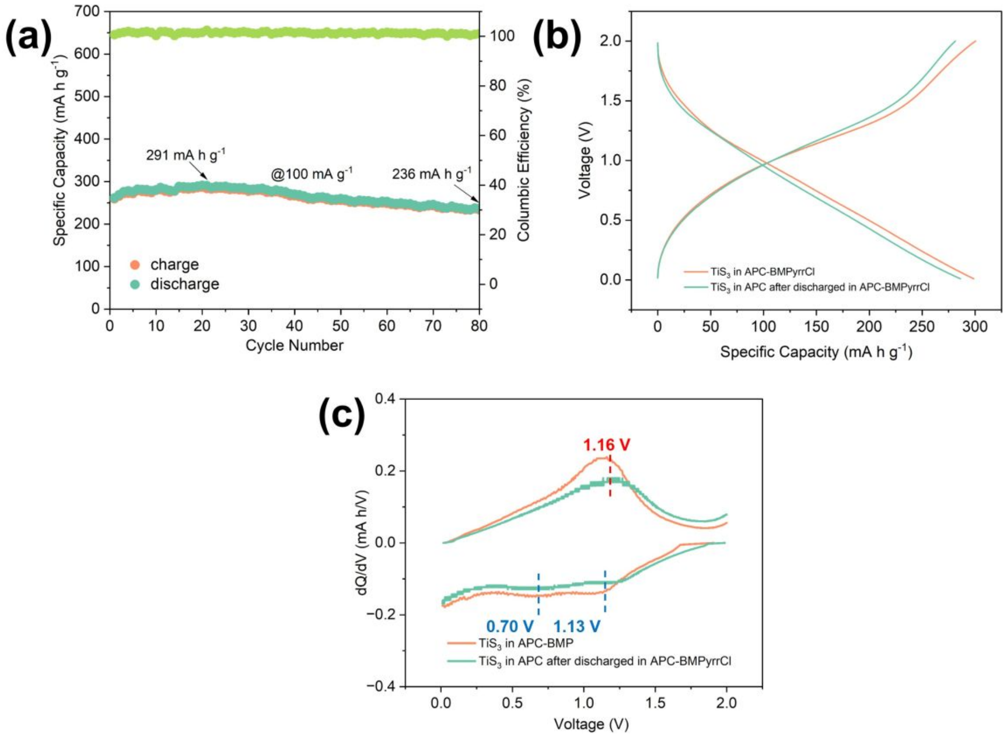

Figure S4. (a) Galvanostatic (dis)charge cycling performance of the "expanded" TiS_3 electrode with *unmodified* APC electrolyte at a current density of 100 mA g^{-1}. Comparisons of the selected (b) (dis)charge curves and (c) corresponding DC curves of the original TiS_3 electrode with $BMPyrr^+$-containing electrolyte (orange curves) and the "expanded" TiS_3 electrode with *unmodified* electrolyte (cyan curves).

As shown in Figures S5a, b, the refined PXRD pattern confirms that the as-synthesised TiS_2 compound crystalises in a trigonal 1T phase (space group: *P*-3*m*1) with cell parameters of a = 3.4045(1) Å and c = 5.6934(1) Å, comparable to the published data (Table S3). SEM image reveals that the powder sample is composed of hexagonal microplates. The cycling experiments show that, at a relatively high current density of 100 mA g^{-1}, the TiS_2 electrode in APC-BMPyrrCl undergoes 365 cycles to reach the maximum capacity of 140 mA h g^{-1} from an initial low capacity of *ca.* 49 mA h g^{-1}, indicating a relatively poor intercalation process (Figure S5c). (TiS_2 in APC electrolyte: a low capacity of less than 20 mA h g^{-1} at a small current density of

24 mA g^{-1}.[2]) The (dis)charge curves and DC curves during the long-term activation process reveals an electrochemical behaviour similar to that reported in TiS_2 studies,[2] yet distinct from that of TiS_3. The cathodic/anodic peaks of the TiS_3 electrode are positioned at higher voltage positions of *ca.* (0.70 V and 1.13 V)/1.16V (Figure S4b, c), while those for the TiS_2 electrode are at *ca.* (0.30 V and 0.98 V)/1.09V (Figure S5d, e). This highlights the role of sulfur anion redox reaction of the TiS_3 electrode in upgrading the output voltage, compared to only titanium centred redox couple Ti^{4+}-to-Ti^{3+} in the TiS_2 electrode.

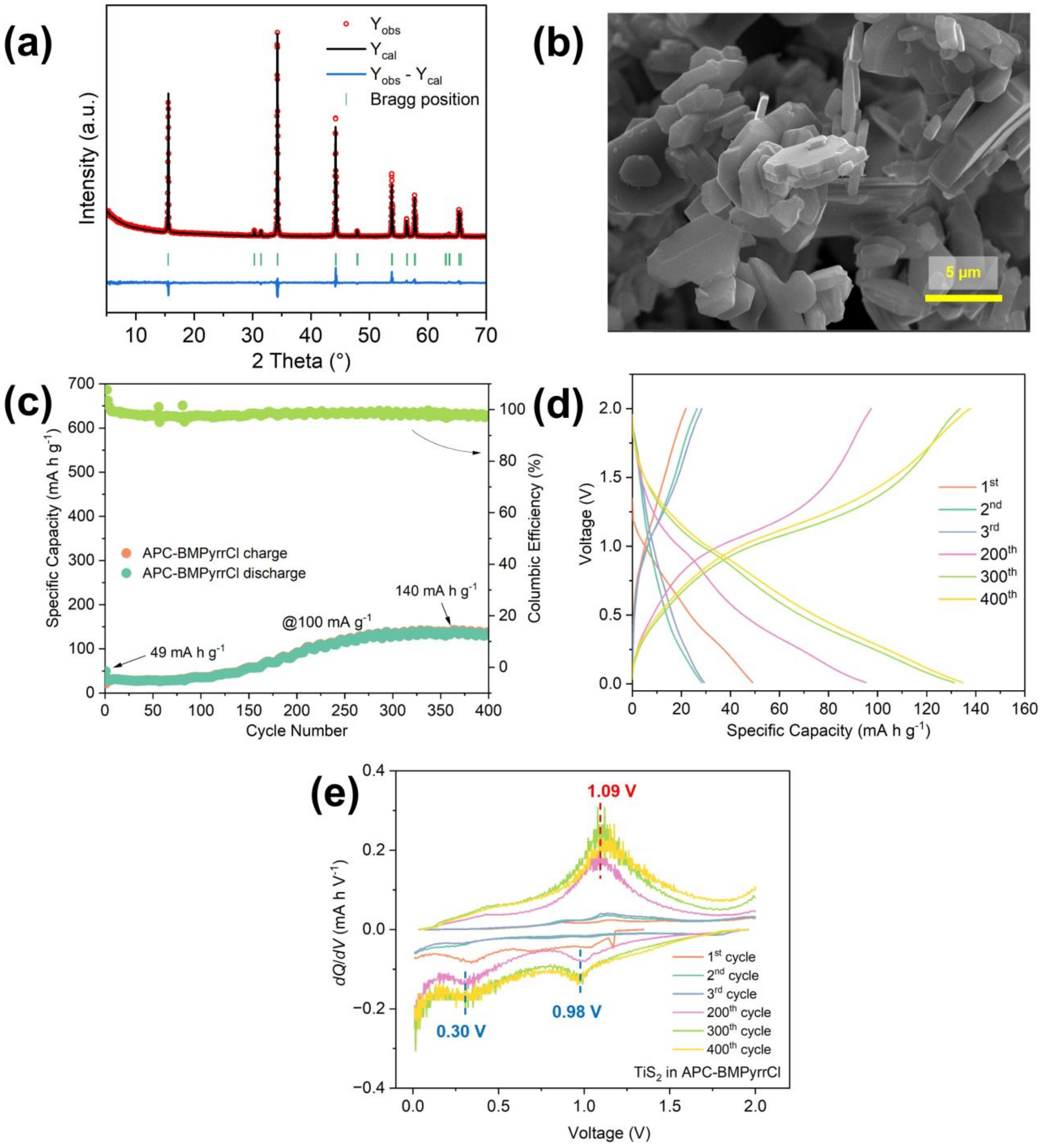

Figure S5. (a) Profile plot of the Rietveld refinement of the measured PXRD pattern for the as-synthesised TiS_2 powder and (b) SEM image of the as-synthesised TiS_2 compound. (c) Galvanostatic (dis)charge cycling performance of the TiS_2 electrode in APC-BMPyrrCl electrolyte at a current density of 100 mA g^{-1}. Selected (d) (dis)charge curves and (e) DC curves from the cycling data.

Table S3. Crystallographic data obtained from Rietveld refinement of the as-made TiS_2 as compared to the published data.[3]

Chemical Formula	Synthesised TiS_2	Published TiS_2
Crystal System	Trigonal	Trigonal
Space Group	*P*-3*m*1	*P*-3*m*1
Lattice Parameter	a = 3.4045(1) Å, c = 5.6934(1) Å	a = 3.404(1) Å, c = 5.696(3) Å
No. of Points	3708	-
N – P + C	3703	-
Formula Weight / g mol^{-1}	111.9	111.9
Calculated Density / g cm^{-3}	3.249	3.25
Volume / Å^3	57.150(2)	57.16
R_{wp}	8.31%	-
R_p	6.43%	-
χ^2	1.95	-

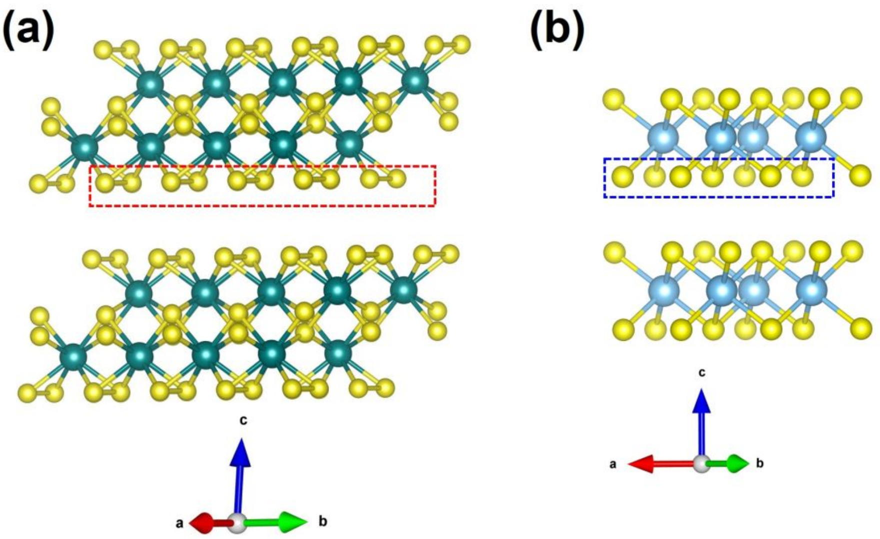

Figure S6. Schematic illustrations of the crystal structures of: (a) quasi-1D pseudo-layered TiS_3 and (b) 2D layered TiS_2.

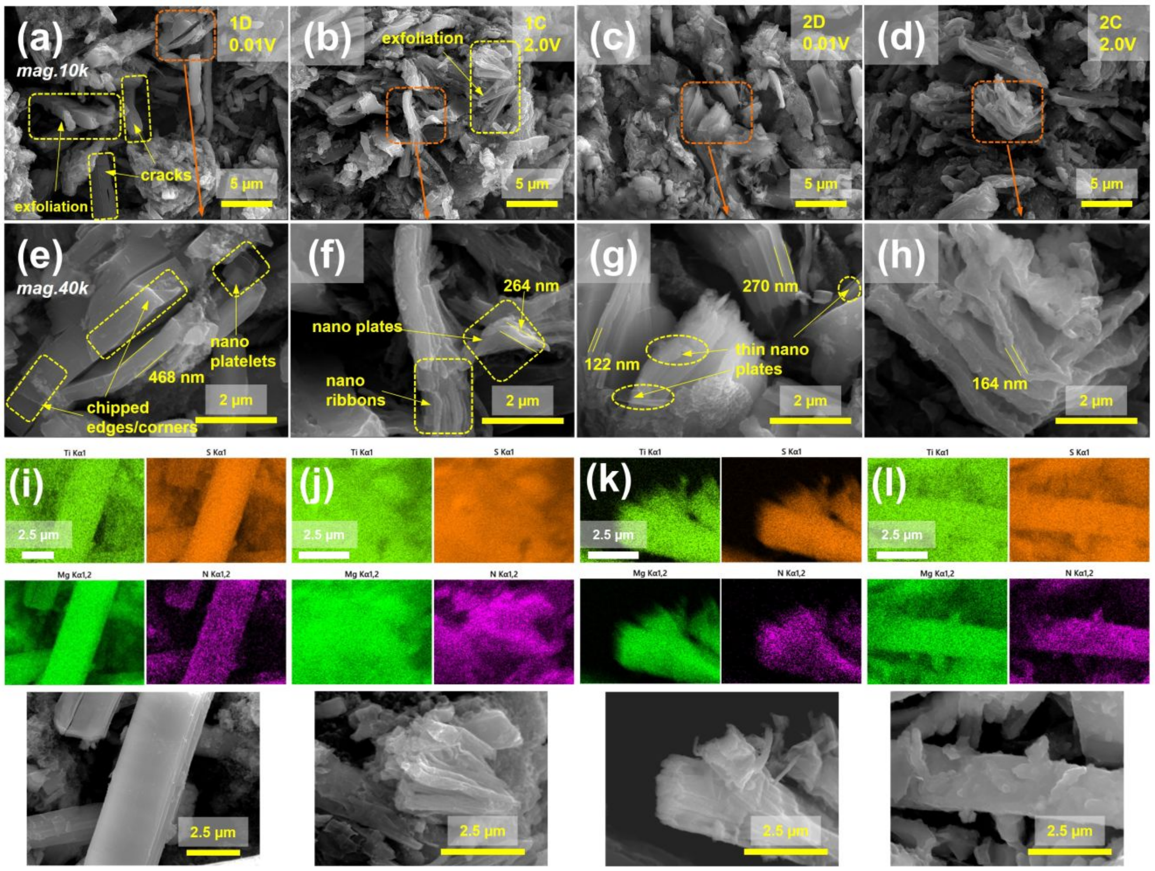

Figure S7. (a) - (h) SEM images (with magnifications of *10k* and 40*k*) and (i) - (l) SEM/EDS elemental maps (Ti, S, Mg, and N) of the electroactive particles at 1D0.01V (a, e, i), 1C2.0V

(b, f, j), 2D0.01V (c, g, k), and 2C2.0V (d, h, l) states of charge. Orange dashed lines and arrows highlight the regions that are magnified to (d)-(f).

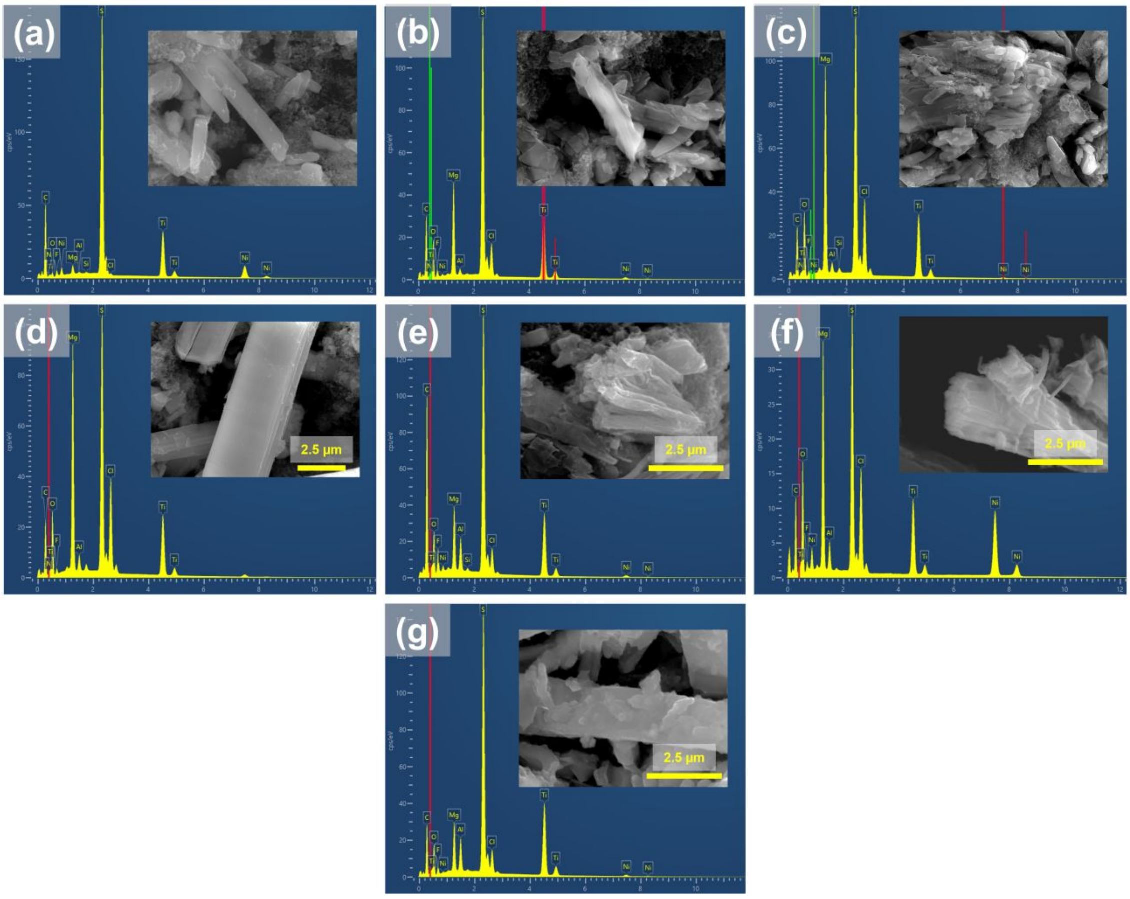

Figure S8. EDS spectra and (inset) corresponding SEM images of the TiS_3 active materials at (a) 1D0.6V, (b) 1D0.5V, (c) 1D0.25V, (d) 1D0.01V, (e) 1C2.0V, (f) 2D0.01V, (g) 2C2.0V states. The EDS spectra were from the elemental maps of Figures 5 and S7.

Table S4. As-detected atomic composition of the TiS_3 electrodes derived from Figure S9 and the corresponding processed atomic ratios of different elements at various (dis)charge states (right, shaded).[a]

As detected (At.%)						Atomic ratios of different elements					
State	Mg	Cl	Al	Ti	S	Mg/Cl	Cl/Al	Mg/Ti	Cl/Ti	Al/Ti	S/Ti
1D0.6V	0.5	0.1	0.1	5.3	14.6	5.00	1.00	0.09	0.02	0.02	2.75
1D0.5V	4.7	1.9	0.3	6.3	13.1	2.47	6.33	0.75	0.30	0.05	2.08
1D0.25V	9.3	4.0	0.4	6.0	11.9	2.33	10.00	1.55	0.67	0.07	1.98
1D0.01V	9.5	4.7	0.7	5.5	11.2	2.02	6.71	1.73	0.85	0.13	2.04
1C2.0V	1.9	0.9	0.9	4.0	7.9	2.11	1.00	0.48	0.23	0.23	1.98
2D0.01V	9.5	3.6	1.0	4.7	8.5	2.64	3.60	2.02	0.77	0.21	1.81
2C2.0V	2.8	1.5	1.7	8.5	14.7	1.87	0.88	0.33	0.18	0.20	1.73

[a] Mg/Ti, Cl/Ti, and Al/Ti are used to compare the quantality of Mg, Cl, Al in the bulk and/or on the surface of the TiS_x particles.

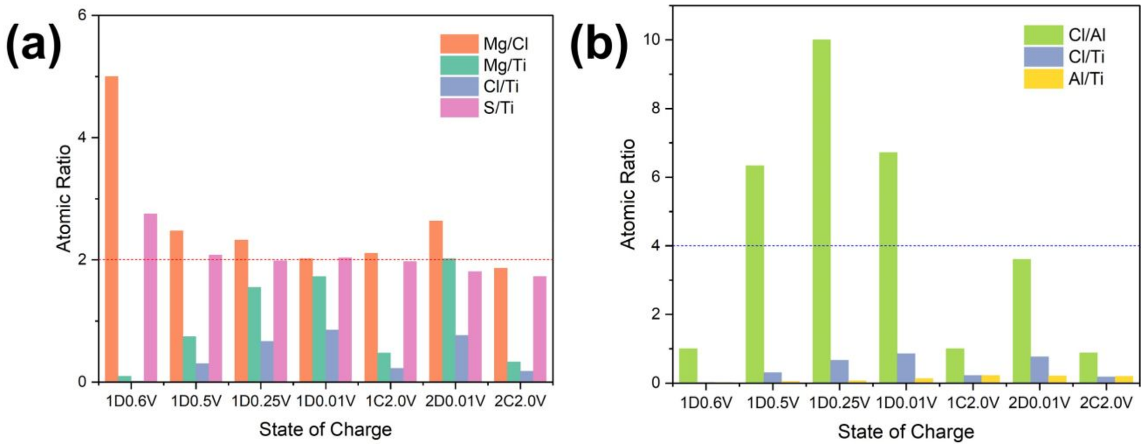

Figure S9. Histogram graph of atomic ratios of (a) Mg/Cl, Mg/Ti, Cl/Ti, and S/Ti, and (b) Cl/Al, Cl/Ti, and Al/Ti in the TiS_3 electrodes at various states of charge.

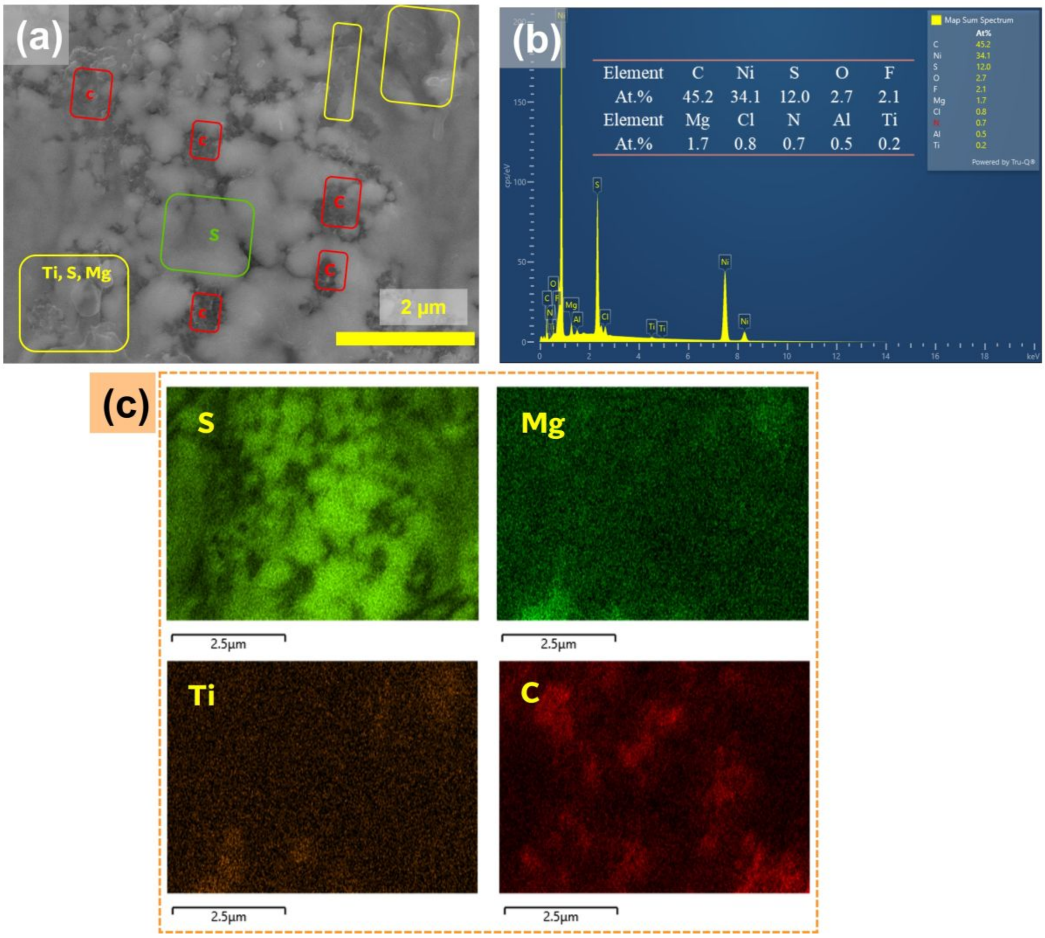

Figure S10. (a) SEM image, and corresponding EDS (b) spectrum and (c) elemental maps of S, Mg, Ti, and C. In (a), regions circled by green, red, and yellow lines indicate S, C, and mixed Ti, S, and Mg.

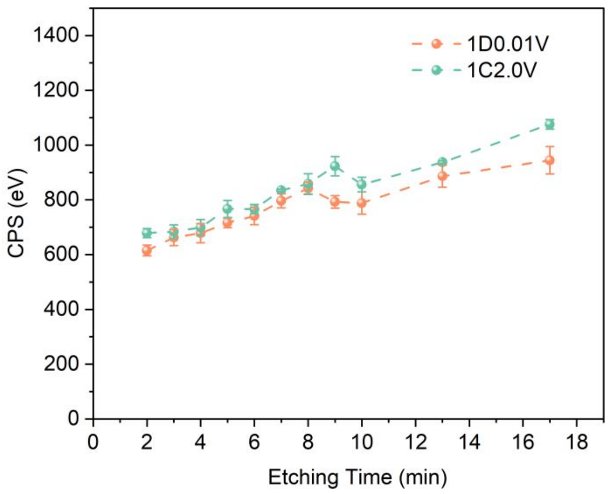

Figure S11. Peak area of N 1s region (indicating the atomic quantity) as a function of etching time at 1D0.01V (orange) and 1C2.0V (cyan) states. A total etching time of 17 min represents an estimated depth of *ca.* 510 nm. The slightly lower $BMPyrr^{+}$ concentration near the surface likely arises from washing and oxidation/amorphous layer. The resulting nitrogen gradient may reflect a transition from the electrolyte absorption layer to the SEI layer, through the surface oxidation or amorphous layer, and into the better-crystallised, ordered titanium sulfide structure at greater depths.

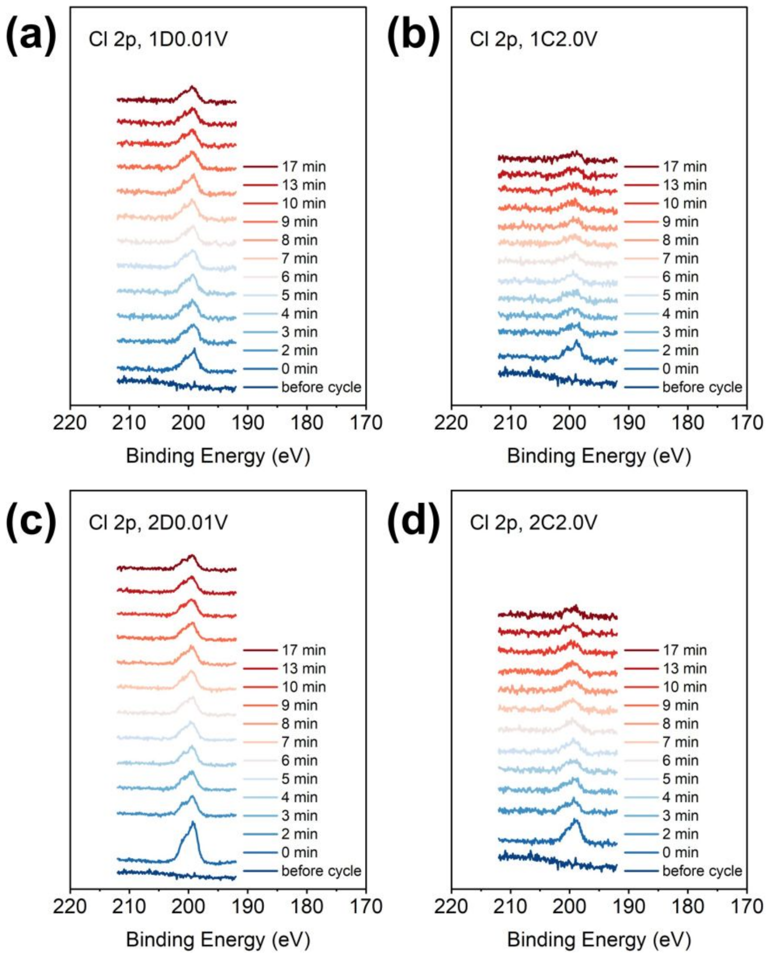

Figure S12. High-resolution XPS spectra of Cl 2p levels of the electrodes at different states of charge with respect to various etching times.

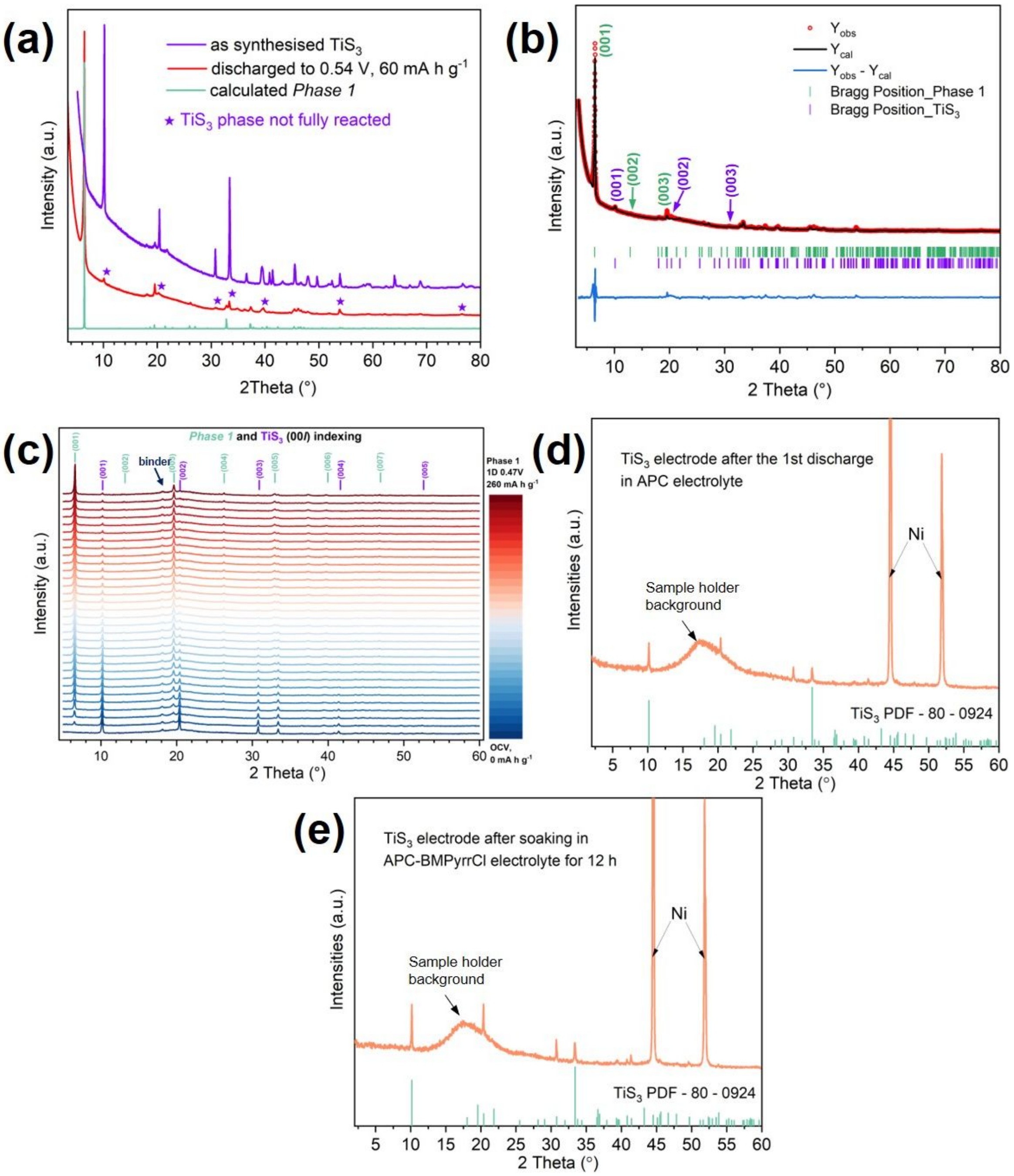

Figure S13. (a) Capillary PXRD patterns of the as-synthesised monoclinic TiS_3 powder (purple), TiS_3 electrode in the 1D0.54 V state (red), and the calculated PXRD pattern (orange) representing *Phase 1* (cyan). The calculated pattern was obtained in Vesta software by increasing the interlayer gap to the assumed value of 1.39 nm through changing the *c* parameter and the *z* coordinates of respective Ti and S atoms in a standard monoclinic TiS_3 unit cell. (b) Profile plot of the Rietveld refinement of the measured PXRD pattern for the electrode in the

1D0.54 V state, using the above expanded monoclinic TiS_3 unit cell as model. (c) Stack plot of the *operando* PXRD patterns of the TiS_3 electrode in selected voltage range from open circuit voltage (OCV) to 0.47 V of the first discharge, with peaks for (00*l*) planes of *Phase 1* and TiS_3 phase indexed based on methods used in (a) and (b). The PXRD patterns of the TiS_3 electrodes (d) discharged in APC electrolyte and (e) immersed in APC-BMPyrrCl electrolyte for 12 h.

Table S5. Crystallographic data of *Phase 1* and TiS_3 phase obtained from the Rietveld refinement of the *ex situ* capillary PXRD pattern for the electrode at 1D0.54 V state.

**Phase**	***Phase 1***	***TiS_3 phase***
Crystal System	Monoclinic	Monoclinic
Space Group	$P2_1/m$	$P2_1/m$
Lattice Parameter	a = 4.959(7) Å,<br>b = 3.384(1) Å,<br>c = 13.813(3) Å,<br>β = 85.4°(2)	a = 4.9571(10) Å, b = 3.4023(7) Å,<br>c = 8.8039(26) Å,<br>β = 97.42°(2)
Volume / $Å^3$	231.1(4)	147.24(6)

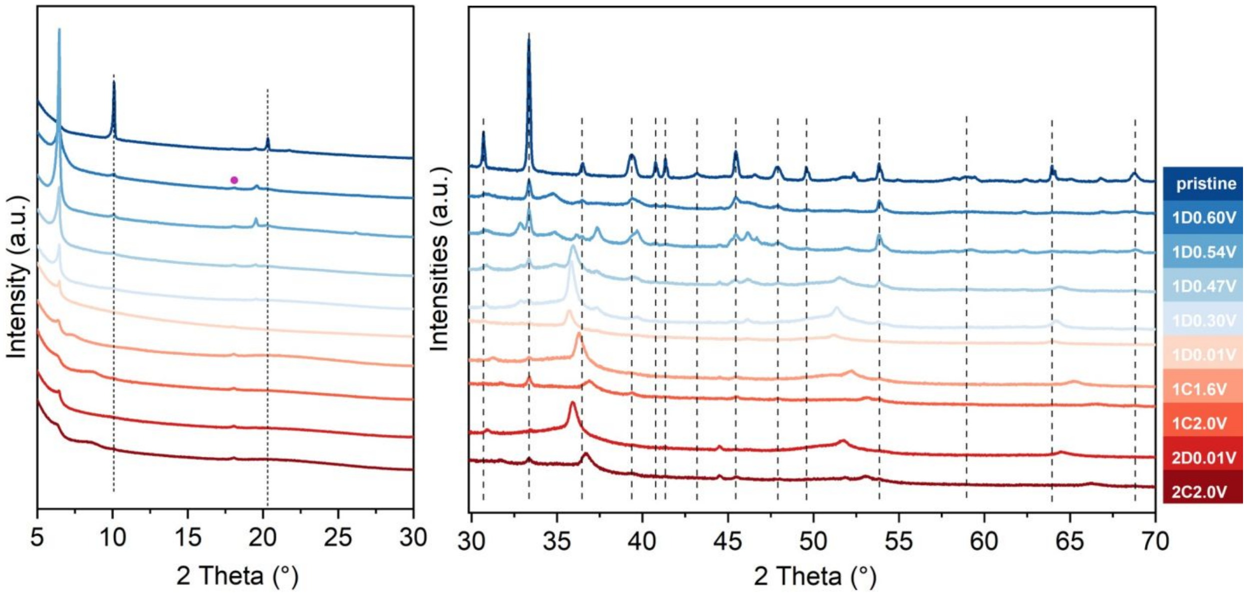

Figure S14. *Ex situ* capillary PXRD patterns (transmission geometry) of the TiS_3 electrodes at different states of charge. The black dashed lines indicate the peak positions for pristine TiS_3. Purple circle indicates the polytetrafluoroethylene (PTFE) binder used for these electrodes.

The CV peak current can be expressed using a power-law relationship, as shown in Equation S1:[4]

$$i = av^b \qquad \text{Equation S1}$$

where i represents the current (mA), v is the scan rate (mV s^{-1}), and a and b are adjustable constants. For pure diffusion-controlled processes, i is proportional to $v^{1/2}$ ($b = 0.5$), while for capacitative processes, i is directly proportional to v ($b = 1$). In systems involving mixed processes, b typically falls between 0.5 and 1.0. A linear fit of log(i) versus log(v) (Equation S2) provides insights into the underlying charge storage mechanisms during electrochemical reactions:

$$log(i) = log(a) + blog(v) \qquad \text{Equation S2}$$

The total current at a specific voltage in CV curves can be described using Equation S3, which separates the contributions from surface-confined capacitive processes (k_1) and bulk

diffusion-controlled processes (k_2). Linear fitting of $i/v^{1/2}$ against $v^{1/2}$ enables the quantification of these contributions:[5]

$$i = k_1 v + k_2 v^{1/2} \text{ or } \frac{i}{v^{1/2}} = k_1 v^{1/2} + k_2 \qquad \text{Equation S3}$$

The diffusion coefficient (D) can be calculated based on Fick's law (Equation S4):[6]

$$D = \frac{4}{\pi\tau}\left(\frac{m_B V_M}{M_B S}\right)^2\left(\frac{\Delta E_S}{\Delta E_\tau}\right)^2 \qquad \text{Equation S4}$$

where τ, is the pulse duration (s), m_B is the mass of the electrode material (g), V_M is the molar volume (cm^3 mol^{-1}), M_B is the molar mass (g mol^{-1}), and S is the electrode area (cm^{-2}). ΔE_τ refers to the potential change during the current pulse without the IR drop (*i.e.* the vertical potential change at the start or end of the current pulse due to resistances),[7, 8] while ΔE_s is the potential difference between equilibrium states before and after the pulse. Both values can be derived from the GITT curves shown in Figure 9d.

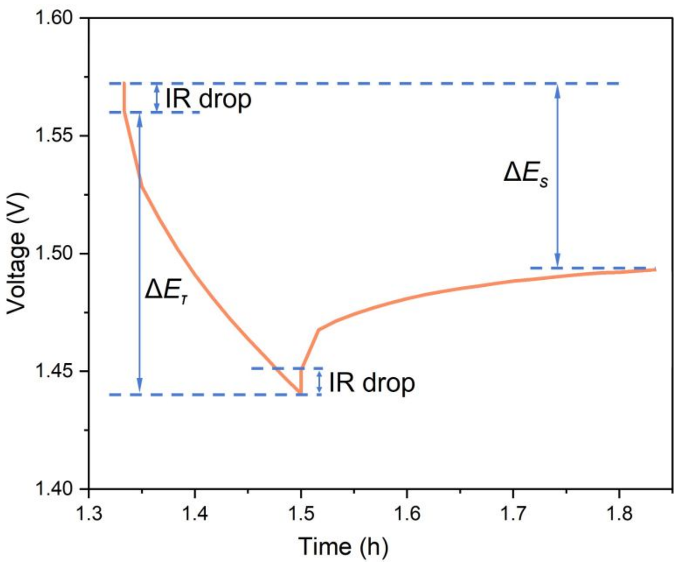

Figure S15. One pair of current pulse and relaxation data taken from the GITT curve of the TiS_3 electrode shown in Figure 9d, illustrating the measurements of ΔE_τ, ΔE_s, and the *IR* drop, respectively.

Table S6. Comparisons of the average diffusion coefficients of TiS_3 electrode with other expanded electrode materials in the literature.

Cathode	Electrolyte	Cell	Experiment conditions	Average diffusion coefficient (cm^2 s^{-1})
**TiS_2 [2]**	APC + BMPyrrCl	Coin-type	24 mA h g^{-1}, RT, 20 min D/C, 30 min R, 0 - 2V	1×10^{-11}
**PAN intercalated-VS_4 [9]**	APC	Coin-type	100 mA h g^{-1}, RT, 5 min D/C, 5 min R, 0.3 - 2.1 V	3.29×10^{-10}
**VMS [8]**	APC + BMPyrrCl	Coin-type	50 mA h g^{-1}, RT, 10 min D/C, 20 min R, 0.2 - 2.0 V	7.59×10^{-11}
**TiS_3 (This work)**	APC + BMPyrrCl	Coin-type	50 mA h g^{-1}, RT, 10 min D/C, 20 min RT, 0.01 - 2.0 V	6×10^{-11}

Footnote: D/C represents discharge/charge current impulse, while R means resting without current impulse.

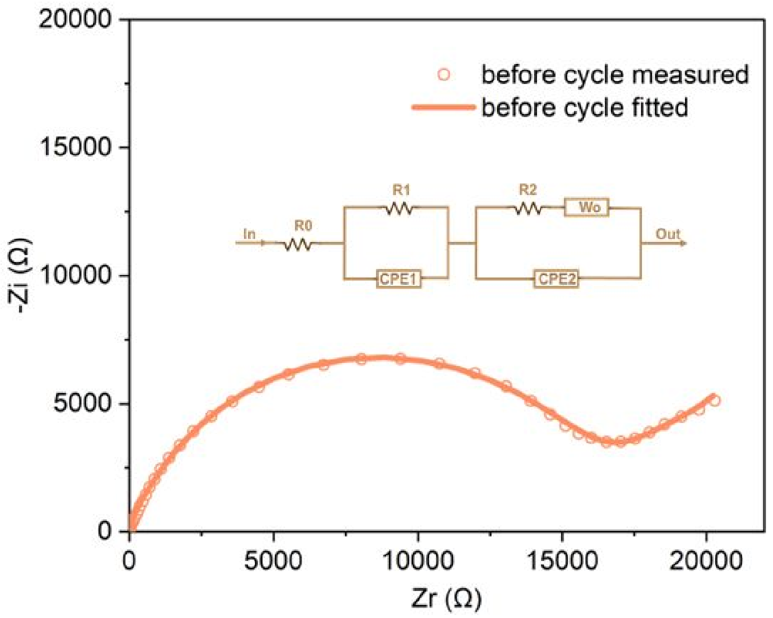

Figure S16. Nyquist plots (hollow circles) and corresponding fit curves (solid lines) of the electrode in Mg|APC-BMPyrrCl|TiS_3 cell before cycle. The equivalent circuit is inset in the graph.

Table S7. Detailed values of parameters derived from the equivalent circuit model of the Mg|APC-BMPyrrCl|TiS_3 cells at various states of charge.

		Q_1(s□ Ω^{-1})	Q_2(s□ Ω^{-1})	R_0(Ω)	R_1(Ω)	R_2(Ω)	W_0(Ω $s^{-0.5}$)
Before cycle	value	3.71E-05	1.10E-05	7.98	2152.10	13444.70	2159.42
	error	6.98E-10	1.73E-07	0.05	15.88	92.88	20.00
1st charge	value	1.05E-05	1.35E-04	8.34	30.77	2835.97	444.06
	error	2.04E-14	1.31E-06	0.00	0.01	27.16	4.38
10th charge	value	1.46E-05	5.55E-04	10.58	30.03	2018.42	349.52
	error	1.54E-10	2.34E-05	0.25	0.79	92.14	7.62

References

(1) Furuseth, S.; Brattås, L.; Kjekshus, A.; Andresen, A.; Fischer, P. On the Crystal Structures

of TiS_3, ZrS_3, $ZrSe_3$, $ZrTe_3$, HfS_3, and $HfSe_3$. *Acta Chem. Scand 10*, 623-631.
(2) Yoo, H. D.; Liang, Y.; Dong, H.; Lin, J.; Wang, H.; Liu, Y.; Ma, L.; Wu, T.; Li, Y.; Ru, Q.; et al. Fast kinetics of magnesium monochloride cations in interlayer-expanded titanium disulfide for magnesium rechargeable batteries. *Nature Communications* **2017**, *8* (1), 339. DOI: 10.1038/s41467-017-00431-9.
(3) Chianelli, R. R.; Scanlon, J. C.; Thompson, A. H. Structure refinement of stoichiometric TiS_2. *Materials Research Bulletin* **1975**, *10* (12), 1379-1382. DOI: https://doi.org/10.1016/0025-5408(75)90100-2.
(4) Ren, W.; Zhang, H.; Guan, C.; Cheng, C. Ultrathin MoS_2 Nanosheets@Metal Organic Framework-Derived N-Doped Carbon Nanowall Arrays as Sodium Ion Battery Anode with Superior Cycling Life and Rate Capability. *Adv. Funct. Mater.* **2017**, *27* (32), 1702116, https://doi.org/10.1002/adfm.201702116. DOI: https://doi.org/10.1002/adfm.201702116 (acccessed 2021/11/06).
(5) Wang, J.; Polleux, J.; Lim, J.; Dunn, B. Pseudocapacitive Contributions to Electrochemical Energy Storage in TiO_2 (Anatase) Nanoparticles. *The Journal of Physical Chemistry C* **2007**, *111* (40), 14925-14931. DOI: 10.1021/jp074464w.
(6) Wen, C. J.; Boukamp, B. A.; Huggins, R. A.; Weppner, W. Thermodynamic and Mass Transport Properties of " LiAl " *Journal of The Electrochemical Society* **1979**, *126* (12), 2258. DOI: 10.1149/1.2128939.
(7) Knehr, K. W.; Biswas, S.; Steingart, D. A. Quantification of the Voltage Losses in the Minimal Architecture Zinc-Bromine Battery Using GITT and EIS. *Journal of The Electrochemical Society* **2017**, *164* (13), A3101. DOI: 10.1149/2.0821713jes.
(8) Jing, P.; Stevenson, S.; Lu, H.; Ren, P.; Abrahams, I.; Gregory, D. H. Pillared Vanadium Molybdenum Disulfide Nanosheets: Toward High-Performance Cathodes for Magnesium-Ion Batteries. *ACS Applied Materials & Interfaces* **2023**, *15* (44), 51036-51049. DOI: 10.1021/acsami.3c10287.
(9) Deng, R.; Wang, Z.; Tan, S.; Lu, G.; Huang, X.; Qu, B.; Huang, G.; Xu, C.; Zhou, X.; Wang, J.; et al. Organic Molecular Intercalation Enabled Anionic Redox Chemistry with Fast Kinetics for High Performance Magnesium Storage. *Small* **2024**, *20* (12), 2308329. DOI: https://doi.org/10.1002/smll.202308329 (acccessed 2024/10/18).
